# Remaining Useful-Life Prediction of the Milling Cutting Tool Using Time–Frequency-Based Features and Deep Learning Models

**DOI:** 10.3390/s23125659

**Published:** 2023-06-17

**Authors:** Sameer Sayyad, Satish Kumar, Arunkumar Bongale, Ketan Kotecha, Ajith Abraham

**Affiliations:** 1 Symbiosis Institute of Technology, Symbiosis International (Deemed University), Pune 412115, India; sameer.sayyad.phd2020@sitpune.edu.in (S.S.); arun.bongale@sitpune.edu.in (A.B.); director@sitpune.edu.in (K.K.); 2 Symbiosis Centre for Applied Artificial Intelligence, Symbiosis International (Deemed University), Pune 412115, India; 3 Faculty of Computing and Data Science, Flame University, Lavale, Pune 412115, India

**Keywords:** feature extraction, milling process, remaining useful life, time–frequency domain, tool wear

## Abstract

The milling machine serves an important role in manufacturing because of its versatility in machining. The cutting tool is a critical component of machining because it is responsible for machining accuracy and surface finishing, impacting industrial productivity. Monitoring the cutting tool’s life is essential to avoid machining downtime caused due to tool wear. To prevent the unplanned downtime of the machine and to utilize the maximum life of the cutting tool, the accurate prediction of the remaining useful life (RUL) cutting tool is essential. Different artificial intelligence (AI) techniques estimate the RUL of cutting tools in milling operations with improved prediction accuracy. The IEEE NUAA Ideahouse dataset has been used in this paper for the RUL estimation of the milling cutter. The accuracy of the prediction is based on the quality of feature engineering performed on the unprocessed data. Feature extraction is a crucial phase in RUL prediction. In this work, the authors considers the time–frequency domain (TFD) features such as short-time Fourier-transform (STFT) and different wavelet transforms (WT) along with deep learning (DL) models such as long short-term memory (LSTM), different variants of LSTN, convolutional neural network (CNN), and hybrid models that are a combination of CCN with LSTM variants for RUL estimation. The TFD feature extraction with LSTM variants and hybrid models performs well for the milling cutting tool RUL estimation.

## 1. Introduction

Machining is an important process in the manufacturing industry [1]. Machining process monitoring plays a vital role in improving the industry’s productivity by reducing unscheduled downtime caused due to failure of the cutting tool [2]. A proper predictive maintenance strategy must be defined to estimate the cutting tool’s life reduced due to tool wear caused during the machining operation [3]. With the evolving artificial intelligence (AI) techniques and advancements in sensor technology, data-driven prediction models are widely used for tool wear and remaining useful life (RUL) prediction [4]. The RUL of a cutting tool is how long it can accomplish the function effectively before it begins considerably deteriorating to perform its purpose. Multiple factors, including the material being cut, the cutting speed, the tool geometry, and the cutting fluid, can affect the RUL of a cutting tool. This paper mainly focuses on the RUL of cutting tools caused by changes in tool geometry due to tool wear. There are numerous approaches for estimating the RUL of a cutting tool, including physics-based models and machine learning (ML) or deep learning (DL)-based methods. Physics-based models, also known as mechanistic models, are based on fundamental physics principles and laws regulating the system. These models seek to describe the behavior of the system using mathematical equations that represent the physical processes involved. In ML/DL models, typically, these methods include monitoring the cutting tool’s condition and performance during use and utilizing this data to anticipate how long the tool can be used before it must be replaced. In the ML/DL-based method, the data-driven approach is an effective technique for forecasting the RUL of the equipment [5].

**Figure 1:**
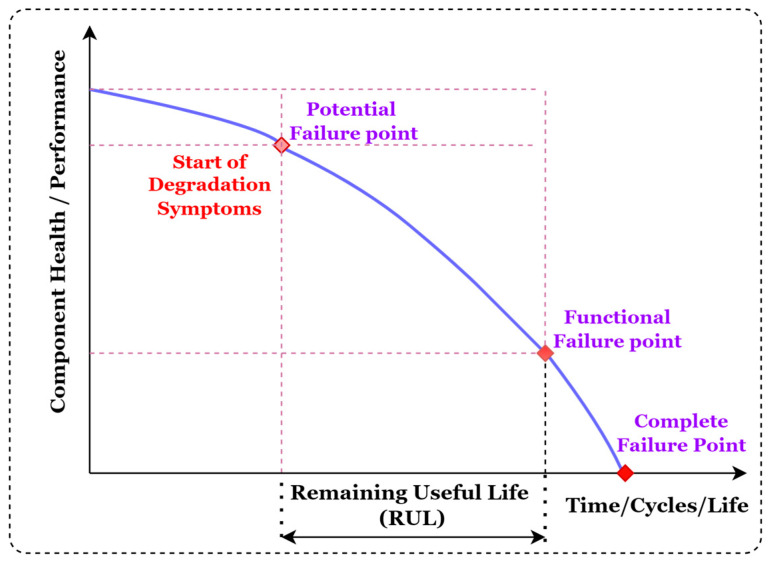
Concept of remaining useful life (RUL) of the equipment.

To prevent the machine’s unplanned downtime and utilize the cutting tool’s maximum life, the accurate prediction of the RUL cutting tool plays an important role [6,7]. Sayyad et al. discussed the effect of unplanned downtime on the equipment cost and profit of the industries [4]. Accurate RUL prediction has the potential to considerably increase the dependability and operational safety of industrial components or systems, thereby reducing the costs of maintenance and preventing severe breakdowns. Figure 1 illustrates the generalized concept of RUL of the equipment.

The prediction accuracy of the RUL estimation plays an important role as accurate prediction helps to utilize the maximum life of the cutting tool. Many researchers aim to predict the tool wear instead of the RUL of the cutting tool. In the case of tool wear prediction, the frequent measurements of the flank or crater wear values are pretty tricky. In manual tool measurement using a tool maker’s microscope, the cutting tool must frequently remove for sensor data and wear mapping, which disturbs the machining process. In comparison, the RUL prediction provides continuous data mapping with sensor data in terms of time. As compared to tool wear as target output, a few research works on the RUL as a target variable in milling cutting tools. Additionally, consolidated comparative studies of the different time–frequency-based feature extraction techniques with various decision-making algorithms are not much addressed in previous works. So, the significant contribution of the work is as follows:

1. To estimate the RUL of the milling cutting tool using the NUAA idea-house dataset [8];
2. To use the time–frequency feature extraction techniques such as STFT, CWT, and WPT to get useful insights from data with reduced data dimensions;
3. To use the different machine learning and deep learning decision-making algorithms for cutting tool RUL prediction and check the performance of each model using different evaluation parameters.

## 2. Related Work

This comprehensive literature analysis aims to investigate the present state of research, various approaches used for RUL estimation in the context of cutting tools, and the different feature extraction and selection techniques used to improve the RUL estimation accuracy. As the physics-based and data-driven driven models are widely used for tool wear and RUL predictions [9,10,11], this section discussed the physics-based and data-driven approaches. Different feature extraction techniques, such as time, frequency, and time–frequency domain and various feature selection techniques, are discussed for RUL prediction.

### 2.1. Physics-Based Model

In physics-based modeling for prediction, fundamental principles and mathematical equations need to be considered to model the system’s behavior. For accurate predictions, a number of factors, such as understanding the system, formulating the equations based on the system’s behavior, assumptions for simplification, estimation of system parameters etc., need to be studied deeply.

In the case of RUL prediction of the cutting tool, the tool geometry, cutting tool, and material materials must be considered. The cutting forces model, degradation mechanism, and wear rate estimation equations are required for estimation; based on this, the health indicators are developed. Model refinement and continuous improvement in the model are required in physics-based modeling to improve prediction accuracy.

Physics-based techniques suffer from a scarcity of accurate analytical models to characterize tool wear processes due to the cutting process’s intrinsic complexity and the machining process’s imperfect understanding [9]. The data-driven modeling approach is preferred in the tool wear and RUL predictions to avoid the modeling uncertainty in the physics-based model [12].

### 2.2. Data-Driven Model

**Figure 2:**
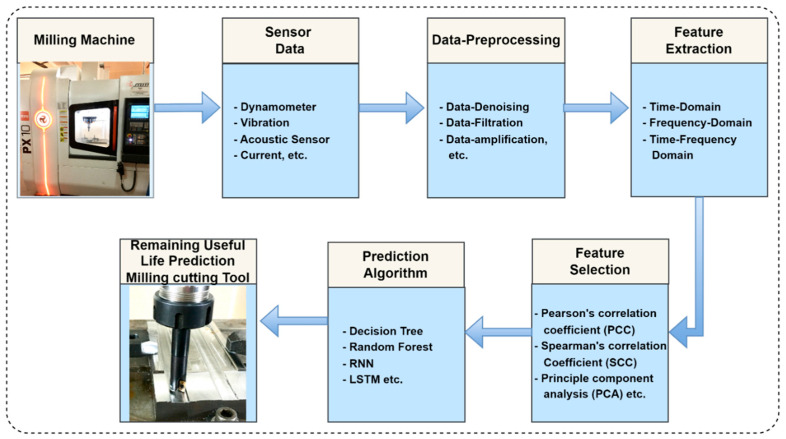
Generalized data-driven model for RUL prediction.

The data-driven makes use of machine learning and statistical approaches to extract patterns and relationships from accessible data. In a data-driven model, the sensor data is collected from the machine by mounting the sensors at the appropriate position to understand the condition of the cutting tool. The dataset should encompass a wide range of tool life spans and operating conditions to capture the variation in tool deterioration patterns. The raw data need to be pre-processed before performing the feature extraction. The data pre-processing ensures the data’s integrity and suitability for modeling. This step entails dealing with missing values, anomalies, and data normalization or standardization to create a consistent scale for the variables. The extracted features from the pre-processed data are provided to decision-making algorithms to get output as the desired RUL prediction value. Figure 2 shows the generalized data-driven model for RUL prediction.

In a data-driven model, the machining signals are gathered using different sensors such as acoustic, dynamometer, current, vibration, etc., using the indirect sensing technique [13,14]. These collected signals are used for RUL prediction by applying feature extraction, feature selection, and prediction algorithms on data.

Many researchers used the data-driven modeling approach for the tool wear and RUL prediction using single or multi-sensors with different feature extraction approaches [15,16,17]. Feature extraction and selection play an essential role in the accuracy of the prediction models in the data-driven models.

### 2.3. Feature Extraction and Selection

Proper feature extraction and selection are crucial in the training phase of any machine and deep learning model. Generally, features are extracted in the time domain (TD), frequency domain (FD), and time–frequency domains (TFD) [18]. TD features mainly represent the change in signal amplitude concerning time. Generally, signals are converted from the TD to the FD spectrum in the FD using the fast Fourier-transform (FFT) technique. The FFT is the sine wave function that provides the transient nature of spectral signal in terms of amplitude and frequency distribution [19]. In the frequency domain, FFT does not consider the abrupt change in the signal. The FD provides the time distribution information in the Fourier-transform (FT) phase characteristics. It is not easy to use this time distribution information of signal in the FD in signal processing [20]. The FFT lacks the ability to provide frequency information over the localized signal region in time. Both TD and FD-based feature extraction techniques are more suitable for stationary signals application where the spectral component of the signal does not change with time [21]. However, most real-time-generated signals are non-stationary types with varied spectral components with time [22]. The TFD feature extraction process is preferred for non-stationary signals generated in the machining process [23]. TFD-based feature extraction techniques mainly include STFT, WT, empirical mode decomposition (EMD), Hilbert–Huang transform (HHT), etc. The STFT and WT, TFD feature extraction techniques provide a good result for tool wear and RUL prediction [23,24,25].

Rafezi et al. use vibration and sound signals to monitor the tool condition in CNC lathe drilling operations [26]. The author uses both TD and TFD features for tool condition monitoring and found that the TFD’s wavelet packet decomposition approach correlates better with tool conditions. Hong et al. use a dynamometer for gathering torque and forces generated during the micro-milling machine [24]. The WPT method extracts the features from the raw signals to monitor the tool wear in micro-milling. Xiang et al. used the accelerometer to capture the vibration signals during milling [27]. To extract features from the input vibration data, the WPT is employed. The extracted WPT features are provided to the backpropagation neural network (BPNN) and LSTM to predict the tool wear class. LSTM shows a higher testing accuracy (up to 95.67%) than the BPNN model for estimating the type of tool wear.

From the literature, it was found that the time–frequency domain feature extraction will provide better prediction results for the non-stationary signals generated during the machining. At the same time, deep learning models such as the LSTM show better prediction results in the time-series data analysis. From the previous work, it was found that limited comparative research has been carried out in the RUL prediction using the TFD feature extraction technique with different feature selection and ML and DL decision-making models. In this work, the time–frequency domain techniques are used for feature extraction with PCC and RF feature selection methods. The various predictions ML and DL models, including SVM, RFR, GBR, LSTM, CNN, LSTM variants, and hybrid models such as CNN with LSTM variants, are used to improve the prediction accuracy of the RUL estimation.

## 3. Time–Frequency Domain Feature Extraction

**Figure 3:**
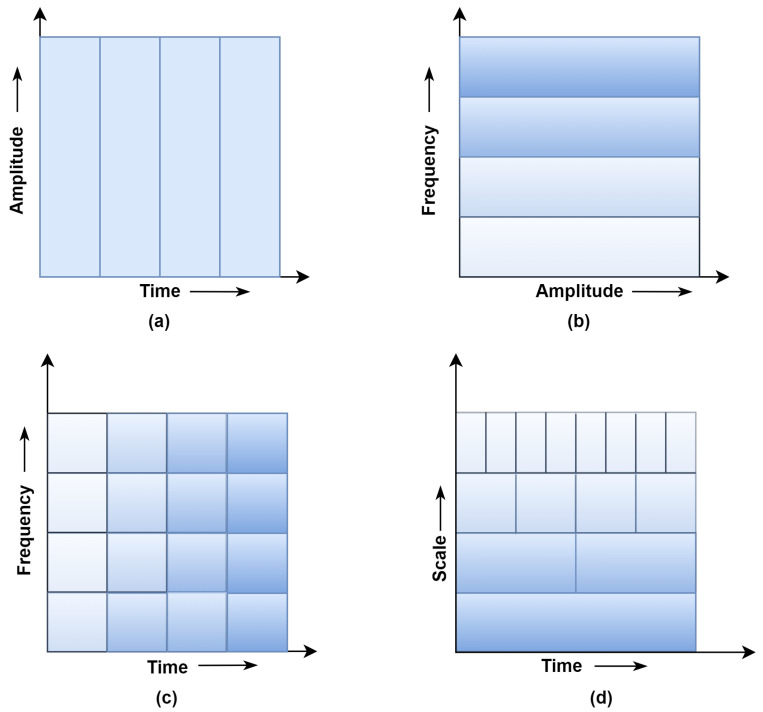
Comparison of windowing approach (**a**) Time-domain, (**b**) Frequency-domain (Fast Fourier Transform) (**c**) Short Time Fourier Transform (STFT) (**d**) Wavelet Analysis.

The non-stationary signals with different time-varying frequency characteristics show poor time-localization in the spectral domain. The TFD analysis is preferred to overcome the TD and FD limitations. Figure 3 shows the signal windowing approach’s comparison of the TD, FD, STFT, and WT [28,29].

In this study, the author used the STFT and WT methods for feature extraction purposes, as these methods show promising results in RUL prediction during machining.

### 3.1. Short-Time Fourier-Transform (STFT)

The poor time-localization problem of the spectral domain’s non-stationary signals is overcome by dividing the original signal into multiple short-duration windows in Fourier-transform; this technique is called window Fourier-transform (WFT) or STFT. FFT does not use the windowing function for signal transformation, as shown in Equation (1). In contrast, for calculating the STFT of the signal, the windowing function is used that is mathematically expressed using Equation (2) [30].

(1) $$F\left(\tau ,\omega \right)=\int_{-\infty }^{+\infty }f\left(t\right)e^{-i\omega t}dt$$

(2) $$S\left(\tau ,\omega \right)=\int_{-\infty }^{+\infty }f\left(t\right)w\left(t-\tau \right)e^{-i\omega t}dt$$

where ‘f(t)’ is the signal to be analyzed, ‘w(t − τ)’ is the window function, ‘τ’ is the translation parameter for time localization, ‘ω’ is the frequency component of the signal.

For computing STFT, different equal-length windowing functions, such as Hamming or Gaussian windows, are used. Discrete Fourier-transform (DFT) is performed on each section separately to form the time–frequency (TF) spectral signal. Reducing window size improves the time resolution resulting in more accurate TF resolution with increased computation time. At the same time, a wide window size results in poor time resolution with good frequency resolutions. The windowing function used in STFT does not vary (not scalable and movable) as the window size chosen before STFT operation.

### 3.2. Wavelet Transforms (WT)

WT is an extension of the FT. WT is the type of TF feature extraction technique. WT uses the family of ‘wavelets’ to decompose the signal. The wavelet is used as a windowing function in WT. Selecting the wavelet family uses different windowing functions such as Symlets, Morlets, Daubechies, Harr, etc. The wavelet functions can be shifted and scaled according to signal requirements. Due to the property of scaling and shifting, WT is adaptable to a wide range of time and frequency resolutions, making it a better alternative to STFT in non-stationary signal analysis. Equation (3) shows the mother wavelet *ψ*(*t*) used to calculate the wavelet transform function [23].

(3) $$\Psi_{z,\tau }\left(t\right)=\frac{1}{\sqrt{z}}\Psi \left(\frac{t-\tau }{z}\right)$$

*ψ*(*t*) = mother wavelet, τ = transformation parameter, z = scaling factor, t = time stamp of generated signal. In the original mother wavelet value of z = 1 and τ = 0. This WT is mainly divided into CWT, DWT, and WPT [31].

#### 3.2.1. Continuous Wavelet Transform (CWT)

CWT is an effective signal transformation technique in stationary and non-stationary signal analysis. The mathematical representation of the CWT of the signal is expressed by Equation (4)

(4) $$CW\left(z,\tau \right)=\frac{1}{\sqrt{z}}\int_{-\infty }^{+\infty }f\left(t\right)\psi^{*}\left(\frac{t-\tau }{z}\right)dt$$

where ‘$f\left(t\right)$’ is the signal for wavelet transform, ‘$\psi^{*}$’ is the complex conjugate of mother wavelet Ψ(t), z is the scaling parameter used for zooming the wavelet, τ is the translation parameter used to define the location of the window. The integral compares the shape of the generated wavelet with the original signal. The equation generates the wavelet coefficient, which shows the correlation between the waveform and generated wavelet used at various scaling and shifting values [32]. However, its computation time is slow and generates redundant signals during its transformation.

#### 3.2.2. Wavelet Packet Transform (WPT)

WPT is an enhancement in DWT. In which both the detailed and approximate coefficient obtained in the DWT is further decomposed at every stage [33].

**Figure 4:**
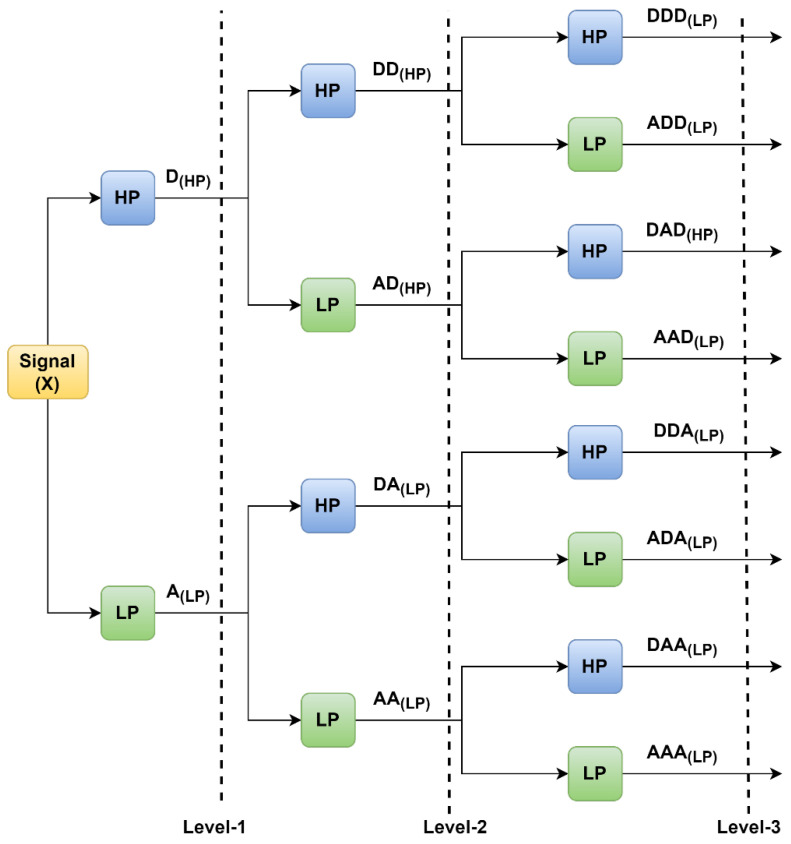
WPT with three levels of decomposition.

Figure 4 shows the WPT with three levels of decomposition. Here, LP and HP are the low-pass and high-pass filters of the signals. The LP and HP are again divided into approximate and detailed coefficients. WPT uses Equation (3) to decompose the signal to calculate the wavelet transform function. WPT uses the two-scale difference to construct scaling and wavelet functions from a single scaling function. The coefficients related to the scaling function, also known as approximation coefficients, are linked with low-frequency data.

In contrast, wavelet function coefficients are correlated with information with high frequency or detail coefficients. Figure 4 shows that 1st level of the decomposition signal is decomposed into D_(HP)_ and A_(LP)_. Similarly, in the second level of decomposition, the approximate signal is decomposed into (AA_(LP)_ and DA_(LP)_), and the detailed coefficient decomposes into DD_(HP)_ and AD_(HP)_.

## 4. Proposed Methodology

**Figure 5:**
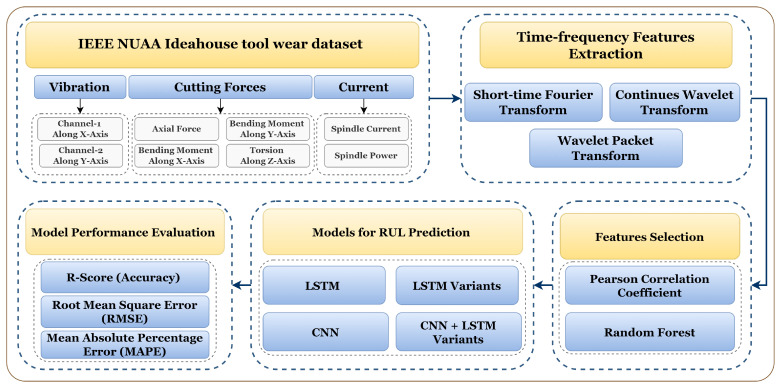
Methodology for tool RUL prediction using different TFD feature extraction methods and LSTM variants.

The overall methodology section is divided into four sub-sections. As the online dataset is used in this work, Section 4.1 discusses the dataset description, Section 4.2 discusses the feature extraction and selection, and Section 4.3 discusses the models used for RUL prediction. Finally, evaluation parameters are discussed that are used for the model comparison. The detailed methodology for RUL prediction is shown in Figure 5.

### 4.1. Dataset Description

**Figure 6:**
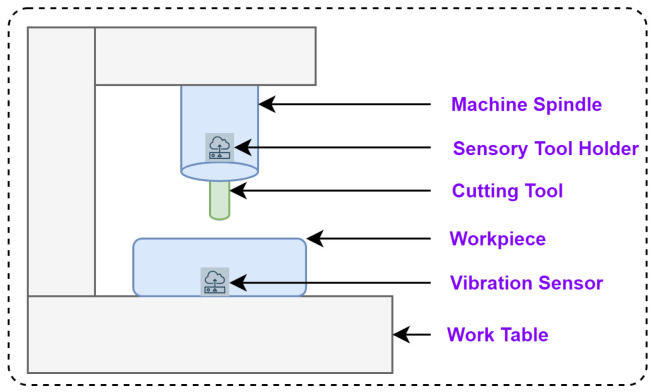
The schematic diagram of the test rig setup for the NUAA Ideahouse dataset.

The IEEE NUAA Ideahouse [8] dataset is used to predict the RUL of the cutting tool. In this dataset, the vibration sensor (PCB^TM^-W356B11), sensory tool holder (Spike^TM^ sensory tool holder), and PLC are used to collect the vibration, cutting forces, and current/power from the milling machine (DMU™ 80P douBlock) during the machining of titanium alloy (TC4) with solid carbide and high-speed steel endmill cutters (12 mm diameter and 75 mm length). Figure 6 shows the schematic representation test rig of the NUAA Ideahouse dataset. The sensory tool holder is connected to the milling machine’s spindle to collect the cutting forces. The vibration sensor is mounted near the workpiece to be machined to collect the vibration signals.

**Figure 7:**
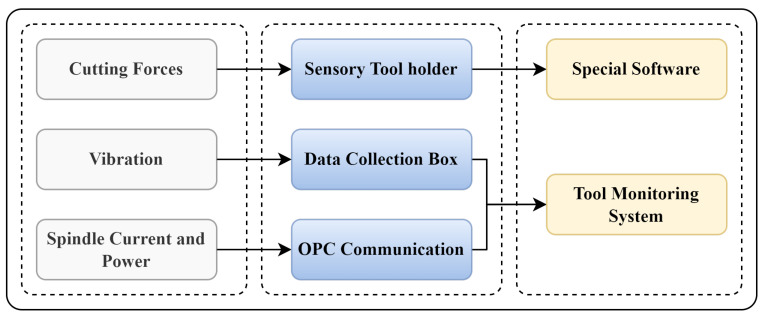
Monitoring signals acquisition for NUAA Ideahouse milling dataset.

Figure 7 shows the signal acquisition system for the NUAA Ideahouse milling dataset. Table 1 shows sampling frequencies for each acquisition equipment. The sampling rates were chosen based on the cutting and spindle speeds. The vibration, cutting forces, and spindle current/power are collected with the sampling rate of 400 Hz, 600 Hz, and 300 Hz, respectively. During the collection process, the low-frequency signals gathered by the software were autonomously interpolated, so the volume of data stored for each signal type is the same. Data synchronization software synchronizes the sampling frequencies at 300 hertz for all the signals.

In the IEEE NUAA Ideahouse [8] dataset, the experiment L9 orthogonal array is created using the experiment design, as shown in Table 2. Out of nine cases, the first two cases, W1 and W2, are considered for the RUL prediction.

A total of thirty runs are taken in case-1 (W1), as shown in Table 3, and the flank wear of the tool is measured after each run. The maximum width of the flank wear is decided based on the ISO-8688 standards. In this dataset, the machining data is collected until the maximum value of the tool wear (maximum flank wear, i.e., VB_max_) reaches up to 0.30 mm. The 0.30 mm is considered the cutting tool’s functional failure during machining in this dataset. The RUL of the cutting tool is estimated based on the value of flank wear. The additional time (in seconds) column is added to the sensor data based on the sampling rate of the data to generate the RUL column for each run. For the W1 run, the maximum value of flank wear is reached up to 0.27 mm. So, all 30 runs are considered for generating the RUL column based on the sampling frequency.

**Figure 8:**
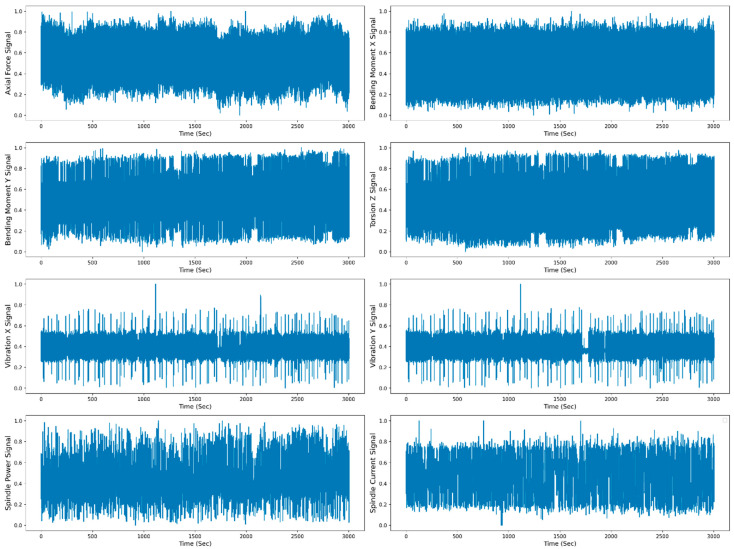
Raw data representation of all the sensor signals to time for the W1 case.

**Figure 9:**
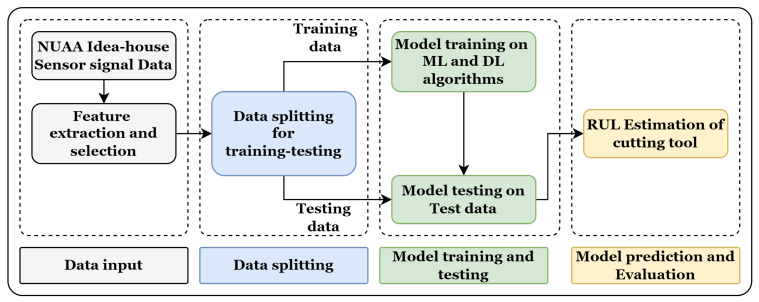
Flowchart of training and testing phases of the RUL prediction approach.

Figure 8 shows the raw data representation (scaled raw data between 0 to 1) of the individual sensor signal with respect to time. For raw data representation, all 30 runs of the W1 case are merged. The total time span to reach the maximum flank tool wear (VB_max_) value from 0 mm to 0.27 mm is 3004 s. The TFD features are extracted from the raw data, and selected features are divided for the model training and testing. The data are split into 70–30% for training and testing. The different ML and DL models are trained on the test data, and the model’s performance is evaluated based on the test data. Figure 9 shows the training and testing phases of the RUL prediction approach.

### 4.2. Feature Extraction and Selection

The raw data in the dataset are normalized and provided for time–frequency feature extraction. The data are extracted in different TFDs such as STFT, CWT, and WPT. The statistical features shown in Table 4 are extracted from the TFD features coefficients vectors.

The extracted statistical TFD features are selected using Pearson’s correlation coefficient (PCC) and random forest regressor (RFR) methods. PCC [34] is extensively used in machining for feature selection in tool wear and RUL prediction. Equation (5) determines the linear correlations between signals and output variables.

(5) $$PCC\left(r\right)=\frac{\sum \left(a_{i}-\bar{a}\right)\left(b_{i}-\bar{b}\right)}{\sqrt{\sum_{i=1}^{n}{\left(a_{i}-\bar{a}\right)}^{2}}\sqrt{\sum_{i=1}^{n}{\left(b_{i}-\bar{b}\right)}^{2}}}$$

where $a_{i}$ = input feature, $\bar{a}$ = average of input feature, $b_{i}$ = target variable, $\bar{b}$ = average of the target variable. The value of “*r*” can range from −1 to 1, with −1 denoting a high degree of negative correlation and 1 denoting a high degree of positive correlation [35].

Another method used for feature selection is the RF method. RF is the embedded feature selection method that lowers the danger of overfitting and performs quicker operations by overcoming the limitations of wrapper and filter feature selection methods [36]. RF is made up of a number of decision trees that were created by randomly extracting characteristics from the data. The significance of a feature is determined by the decrease in impurity or the increase in node purity that results from dividing a specific feature. Whenever a division is made during the building of each decision tree, the decrease in impurity is noted. This decrease is accumulated for every feature across the entire forest. The final step is to normalize the accumulated diminution by dividing it by the total number of trees, providing the feature importance score. The model creates a set containing the necessary features by trimming trees below a given node. The selected features using PCC and RF methods are provided for different RUL prediction models.

### 4.3. Models for RUL Prediction

The different models are used for RUL prediction, including ML models such as SVM, RFR, and GBR and DL models such as LSTM, LSTM variants, CNN, and hybrid models, which combine CNN and LSTM variants. This section discussed the brief about the LSTM, LSTM variants, and CNN with the LSTM model.

**Figure 10:**
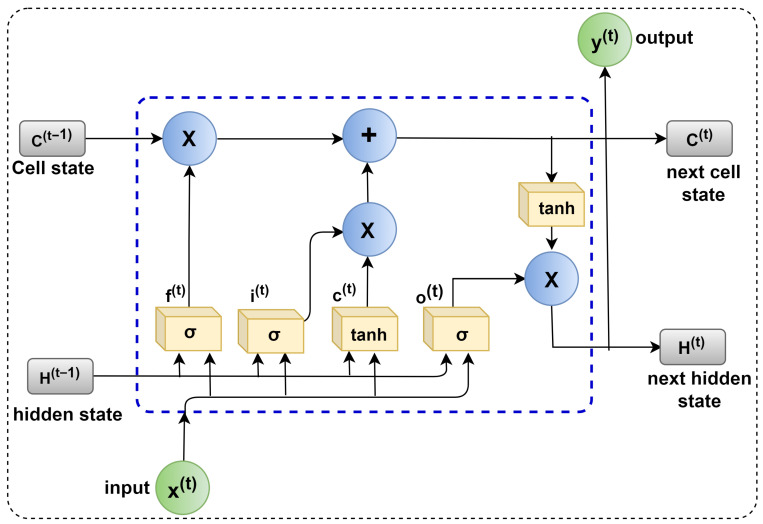
The architecture of the LSTM unit.

The LSTM [37] shows promising performance in tool wear and RUL prediction [38,39,40]. LSTM is an advancement of the recurrent neural network (RNN) [41]. The RNN’s gradient vanishing drawback was reduced in the LSTM structure [42]. The architecture of an LSTM unit is depicted in Figure 10. Long-range dependencies are exploited due to the improvements in the LSTM.

The LSTM modifies the memory at each step rather than overwriting it. The LSTM’s main component is the cell. To add or change cell memory, the LSTM employs sigmoidal gates. ‘Input gate-I’, ‘candidate gate-C’, ‘output gate-O’, and ‘forget gate F’ make up a sigmoidal gate. A(t − 1) and A(t) denote the memory of the previous and subsequent units, respectively. The previous and next cell is hidden state outputs represented by B(t) and B(t − 1). X(t) is the input value, whereas X is element-wise multiplication. The Y(t) indicates the output generated by the LSTM cell. The next unit cell is updated by the gate parameters by modifying or adjusting the parameters and filtering the information. Le et al. [43] discussed the detailed working of the LSTM model.

**Figure 11:**
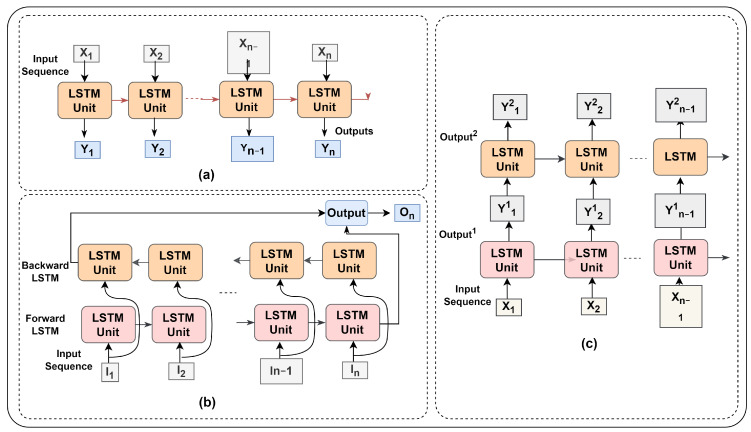
LSTM variants. (**a**) Normal (Vanilla), (**b**) Bi-Directional, and (**c**) Stack.

Figure 11 depicts several LSMT model versions. The vanilla LSTM comprises a single hidden layer of LSTM units that can only access sequential data in one way [44]. The stack LSTM model, on either hand, considers the many hidden LSTM layers. Whereas the forward and backward LSTMs are combined to form the Bi-directional LSTM. The architecture of different LSTM versions is also discussed by Kolekar et al., Chandra et al., and Zhao et al. [45,46,47].

**Figure 12:**
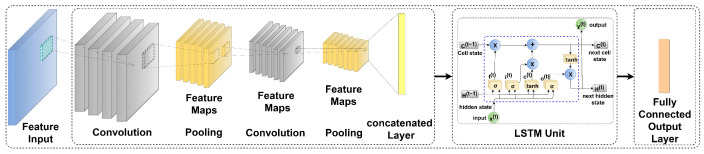
Hybrid CNN-LSTM architecture for RUL prediction.

Figure 12 shows the combination of the CNN-LSTM architecture [48] for the RUL prediction of the cutting tool. Zhang X et al. and Agga A et al. discussed the architecture of the CNN-LSTM in detailed [48,49]. In this work, along with the CNN-LSTM, the different variants of the SLTM are combined with the CNN model, such as CNN-Vanilla LSTM, CNN-Bidirectional LSTM, and CNN-stack LSTM.

### 4.4. Performance Evaluation Parameters

Different performance measurement parameters, such as ‘R-squared score (R^2^)’, ‘Root Mean Square Error (RMSE)’, and ‘Mean Absolute Percent Error (MAPE)’, are used to measure the extent to which these prediction models work. The R^2^ is a metric that assesses the accuracy of a forecast based on real and predicted data [50].

It is used to evaluate the regression model performance by determining how far the predicted points are from the actual data points. Whereas the RMSE provides the square root of the average of predicted and actual values. Finally, MAPE is used to calculate the percentage prediction errors. The formulae for all the performance parameters are provided in Table 5, where *n* = number of data points, ${\ddot{a}}_{i}$ = predicted value, and $a_{i}$ = true or actual value.

## 5. Results and Discussion

The NUAA Ideahouse dataset is in raw signal format with eight incoming signals, including four cutting forces, two vibrations, and one current and power signal, as mentioned in Section 4. From the L9 orthogonal array, the first two cases, W1 and W2, are considered in this work. The results related to case W1 are thoroughly elaborated in this section, and the summarized results table is provided for the W2 case at the end of the result section.

The time column is added to the dataset based on the sampling frequency (300 Hz/per signal). The actual values of the RUL are calculated based on the time column. The features are extracted and selected based on the sensor data as input and RUL as a target feature for the RUL prediction models. The data are normalized using the z-score data normalization technique before passing them to the model. This result section is organized into three parts, i.e.,:

1. The feature extraction based on different TFD techniques such as CWT, STFT, and WPT and feature selection using PCC and RFR methods is discussed;
2. Model performance for each TFD feature using PCC and RF feature selection techniques using different ML (SVM, RFR, and GBR) and DL models (LSTM, LSTM variants, CNN, and hybrid model consisting of CNN with LSTM variants) are evaluated;
3. Finally, the graphs indicating the actual and predicted RUL of the cutting tool versus the actual machining time of milling are plotted for each condition, and a summary of all the obtained results is discussed.

### 5.1. Feature Extraction and Selection

The features are extracted in the TFD using STFT, CWT, and WPT. The statistical features are extracted from the generated time–frequency coefficient vectors. A total of 64 features are extracted in STFT and CWT each, as the number of input signals is eight (Figure 5), and eight statistical features (Table 3) are generated from each signal. In the WPT, the extracted coefficients are divided into approximate and detailed coefficients, generating a total of 128 features (64 approximate and 64 detailed). The extracted features in each method are shown in Table 6.

After feature extraction, the features are selected using PCC and RF methods. In PCC, features with a correlation greater than 0.2 are chosen, whereas in RF, features with a weightage greater than 0.5 are chosen. The selection of threshold values for PCC and RF is finalized after many iterations. The threshold values are kept constant for all the feature extraction techniques to compare model performance.

**Figure 13:**
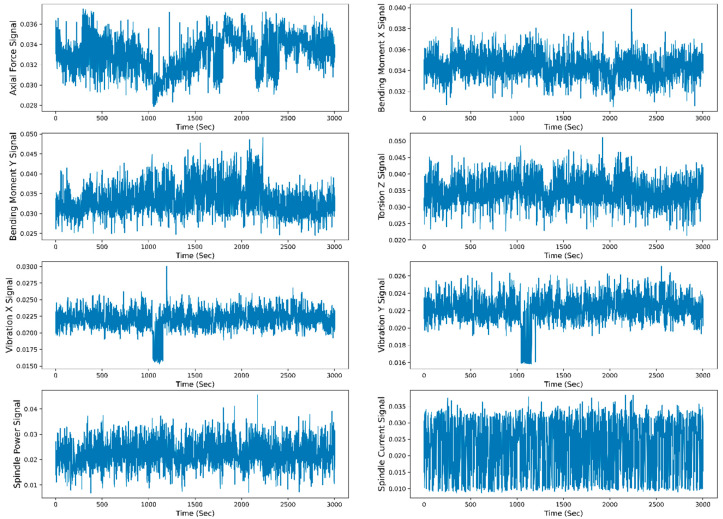
Extracted mean STFT sensor signals representation with time.

Figure 13 shows the change in the mean STFT representation of the individual sensor signal with respect to time. Table 7 shows the selected features using the PCC feature selection technique for extracted STFT-based features. The feature names are indicated by the type of feature extraction technique followed by the type of statistical feature considered and the signal considered for feature extraction. A total of twenty-one features are selected that are having correlation coefficient greater than 0.2. Similarly, Table 8 shows the selected features using RF for STFT feature extraction. Out of 64 features, 31 high-weightage features are selected.

**Figure 14:**
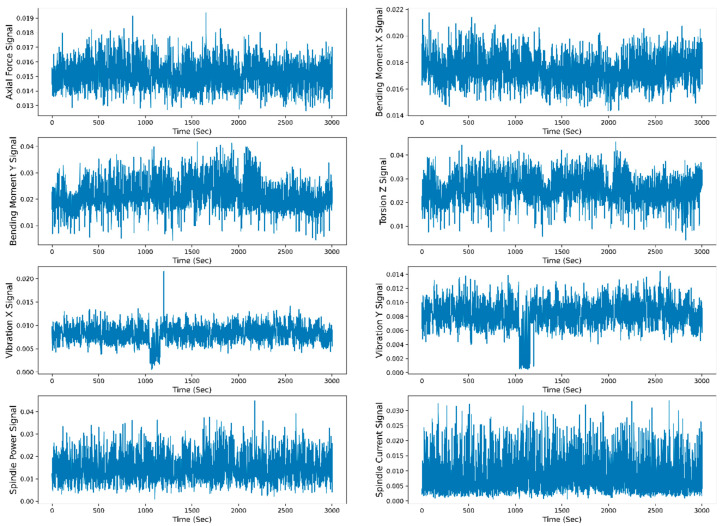
Extracted mean CWT sensor signals representation with time.

Figure 14 shows the change in the mean CWT representation of the individual sensor signal with respect to time. Table 9 and Table 10 indicate the feature selected using PCC and RF from extracted CWT features. The eleven features having a PCC value higher than 0.2 are selected for prediction model training and testing. Forty-three features are selected based on RF weightage greater than 0.5.

**Figure 15:**
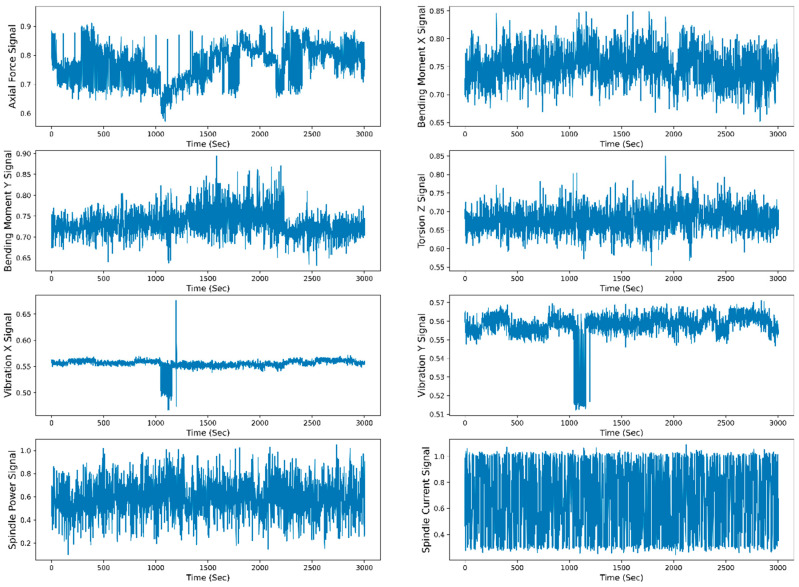
Extracted mean WPT sensor signals representation with time.

Figure 15 shows the change in the mean WPT representation of the individual sensor signal with respect to time. Table 11 indicates the selected 26 features using the PCC technique from the extracted 128 WPT features at the first level of decomposition. The ‘a’ and ‘d’ indicate the extracted approximate and detailed feature coefficients, followed by the extracted statistical details and signal names.

Table 12 indicates the selected features using the RF feature selection method from extracted WPT features. A total of 19 features are selected, with a weightage greater than 0.5.

### 5.2. Machine Learning Models Performance

The extracted and selected features are initially provided to different machine learning (ML) algorithms to check each model’s performance for RUL prediction. Various approaches for selecting features, including PCC and RFR methods, are used to assess the efficacy of each prediction model. In ML models, support vector machine (SVM), random forest regressor (RFR), and gradient boosting regressor (GBR) are used for RUL prediction.

Table 13 shows the performance evaluation for the different ML models using the PCC feature selection technique. The RUL prediction based on the ML model performs poorly compared to DL algorithms. The maximum value of R^2^ is 0.366 for the PCC-based feature selection method given by the RFR model for WPT feature extraction. Whereas, for the same extracted feature, the features are selected using the RF, and the performance of the ML models is slightly improved, as shown in Table 14. The WPT shows the maximum R^2^ of 0.496 for the RFR model in the RF base features selection method. The different DL models, such as LSTM, LSTM variants, CNN, and a combination of CNN with different LSTM variants, are used to improve the performance of the prediction models.

### 5.3. Deep Learning Model Performance

In the DL models, the extracted and selected features are initially provided to the different LSTM variants, such as Vanilla, Bi-directional, and Stack LSTM models, to check each model’s performance for RUL prediction. Similarly, the selected features are passed to the CNN model along with the hybrid model of CNN with different LSTM variants.

In this work, the call-backs and early-stopping approach are used to increase the performance and efficiency of DL models. Call-backs are functions that can be set to execute at certain points during training, such as after each epoch or after a given number of batches have been processed. These capabilities can be utilized to carry out a range of operations, including altering the learning rate, tracking training progress, and preserving model checkpoints, whereas early stopping, on the other hand, is a technique that is used to prevent overfitting. A call-back that checks the validation performance at the end of each epoch and stops training if the performance has not increased for a given number of epochs can be used to enable early stopping in deep learning models. These two approaches increase the effectiveness of the training process by preventing overfitting, conserving time, and reducing the amount of computational resources needed.

In this work, different performance evaluation parameters, such as R-squared (R^2^), RMSE, and MAPE, are considered to check the performance of each model. Generally, in regression, the R^2^ values above 0.90 and MAPE values below 10% are considered models showing good prediction values.

#### 5.3.1. RUL Prediction Using STFT Feature Extraction Technique

The RUL of the cutting tool is predicted using the STFT feature extraction technique. Table 15 shows the performance evaluation parameters of the different LSTM variants model using STFT time–frequency-based feature extraction techniques for RUL prediction. For the PCC-based feature selection technique, the stack LSTM shows the maximum testing accuracy of 0.802, with 0.125 and 7.372% as RMSE and MAPE values, respectively. In RFR feature selection, stack LSTM provides a maximum R^2^ score value of 0.782 as testing accuracy with 0.131 RMSE value and 08.52% MAPE value.

**Figure 16:**
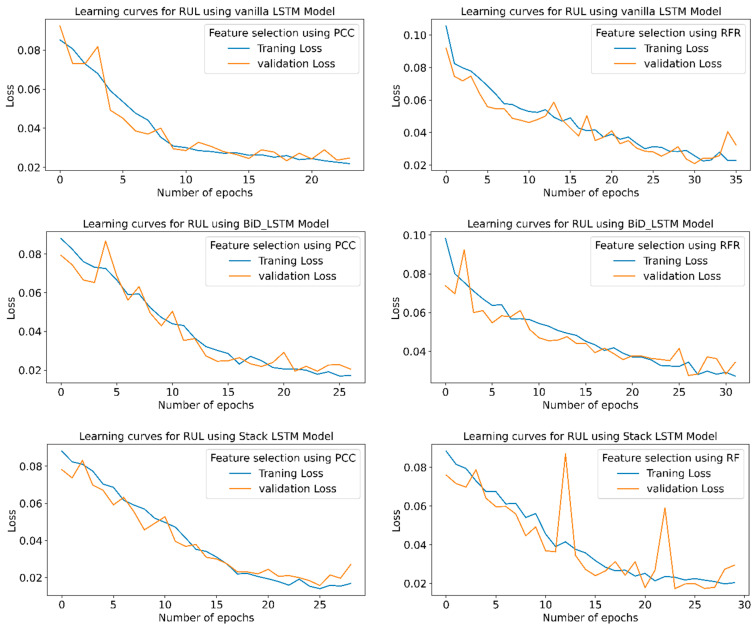
RUL prediction learning curves using STFT-based feature extraction for different LSTM variants.

Figure 16 shows the learning curves for the RUL prediction, indicating the loss vs. the number of epochs for each model using PCC and RFR feature extraction in the STFT feature extraction technique. The graph shows that the losses are minimum for the highest R^2^ and minimum RMSE or MAPE values. Stack LSTM model offers a minimum loss for the PCC and RFR-based feature selection.

**Figure 17:**
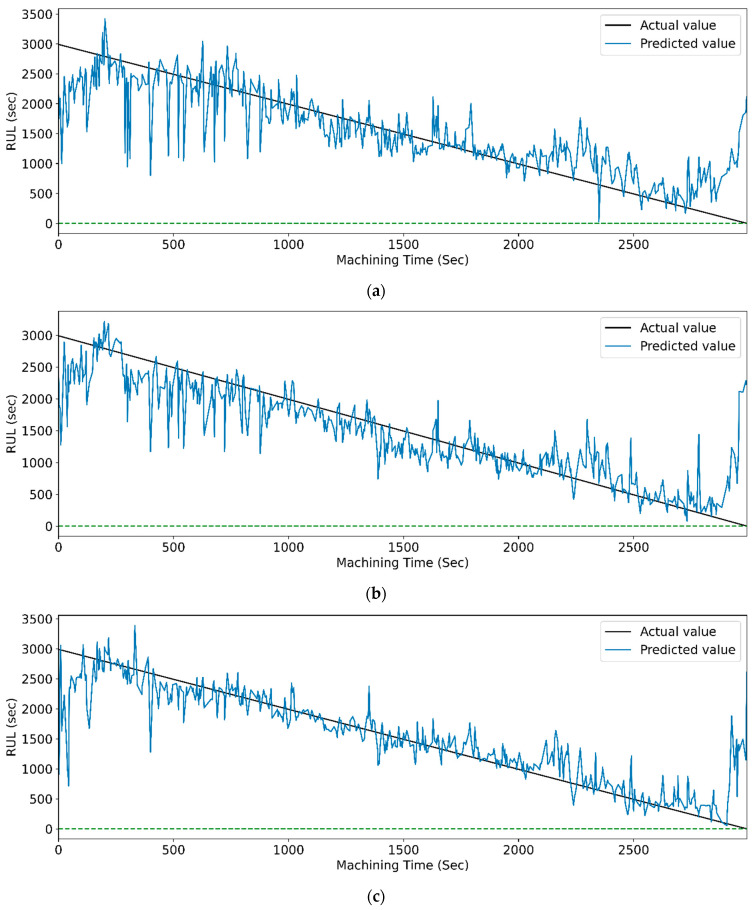
The actual and predicted value of RUL versus machining time for STFT and PCC-based feature selection using different LSTM variants. (**a**) Vanilla. (**b**) Bi-directional, and (**c**) Stack.

**Figure 18:**
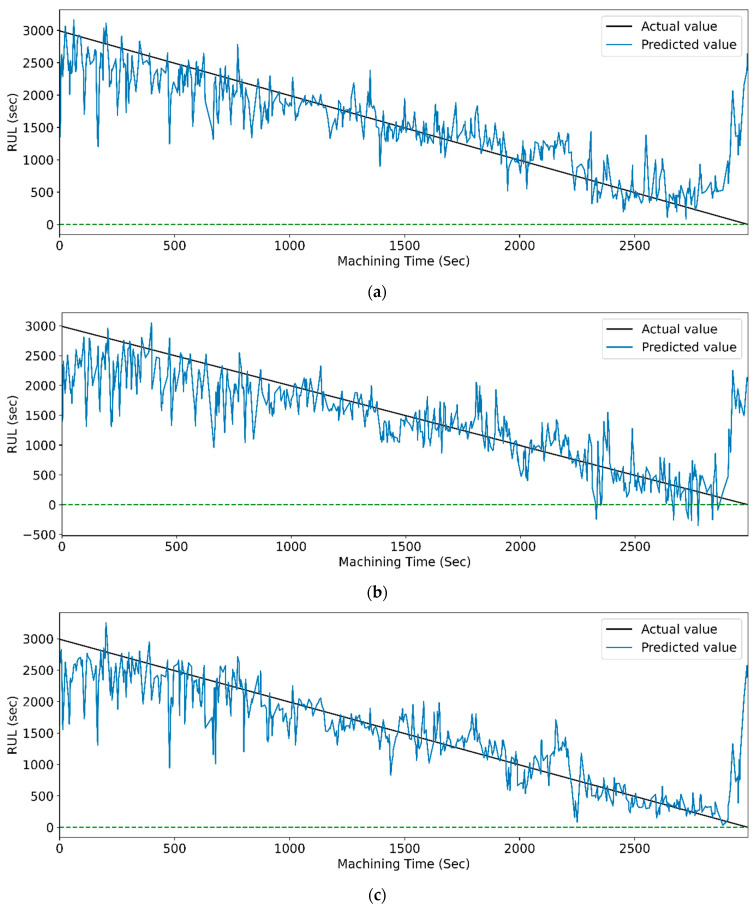
The actual and predicted value of RUL versus machining time for STFT and RFR-based feature selection using different LSTM variants. (**a**) Vanilla, (**b**) Bi-directional, and (**c**) Stack.

Figure 17 and Figure 18 show the graphs of the actual and predicted RUL of the cutting tool concerning total machining time for PCC-based and RFR-based feature selection. The stack LSTM shows the minimum deviation in RUL prediction for both feature selection techniques.

Similarly, Table 16 shows the performance evaluation parameters of the CNN and CNN-LSTM variants models using STFT time–frequency-based feature extraction techniques for RUL prediction. For the PCC-based feature selection technique, the CNN-LSTM shows the maximum testing accuracy of 0.881, with 0.097 and 6.877% as RMSE and MAPE values, respectively. In RFR feature selection, CNN-bidirectional LSTM provides a maximum R^2^ score of 0.951 as testing accuracy with 0.062 RMSE value and 04.161% MAPE value.

**Figure 19:**
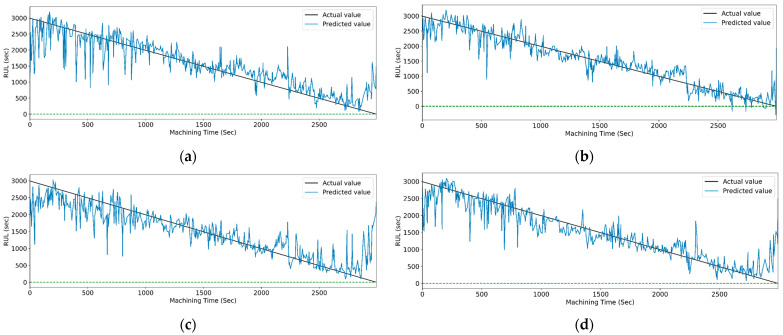
The actual and predicted values of RUL versus machining time for STFT and PCC-based feature selection using different models. (**a**) CCN, (**b**) CNN-LSTM, (**c**) CNN-bidirectional LSTM, and (**d**) CNN-Stack LSTM.

**Figure 20:**
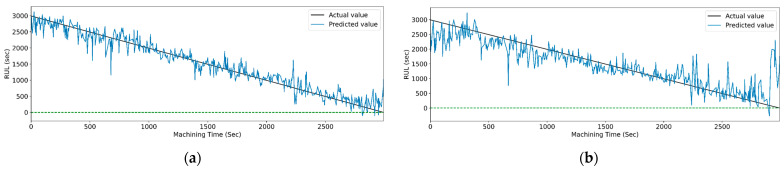

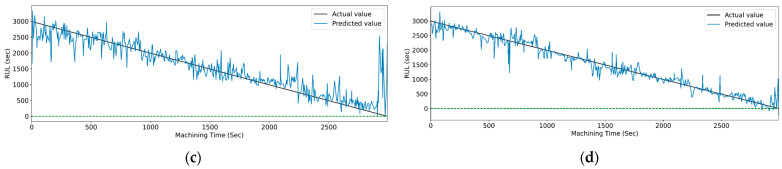
The actual and predicted value of RUL versus machining time for STFT and RF-based feature selection using different models. (**a**) CCN, (**b**) CNN-LSTM, (**c**) CNN-bidirectional LSTM, and (**d**) CNN-Stack LSTM.

Figure 19 and Figure 20 show the actual and predicted values of the RUL for the PCC-based and RF-based feature selection techniques, respectively. In PCC-based feature selection, as the CNN-LSTM shows the maximum accuracy, Figure 19b shows the minimum deviation between the actual and predicted RUL values. Similarly, in RFR-based feature selection, Figure 20d, the CNN-Stack-LSTM shows the minimum deviation in actual and predicted RUL values with maximum accuracy.

#### 5.3.2. RUL Prediction Using CWT Feature Extraction Technique

Table 17 shows the performance evaluation parameters of the different LSTM variants model using CWT time–frequency-based feature extraction techniques for RUL prediction. In CWT, for vanilla LSTM, the maximum testing accuracy is 0.851, with 0.104 and 7.359 as RMSE and MAPE values, respectively. In RFR feature selection, stack LSTM provides a maximum R^2^ score value of 0.927 as testing accuracy with 0.075 and 5.781 as RMSE and MAPE values, respectively.

**Figure 21:**
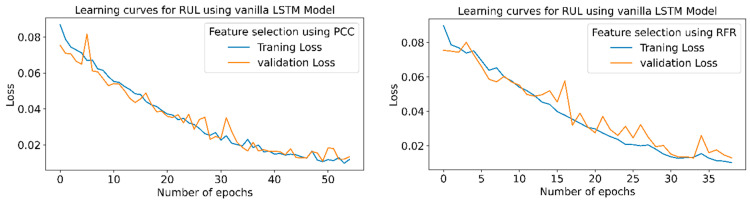

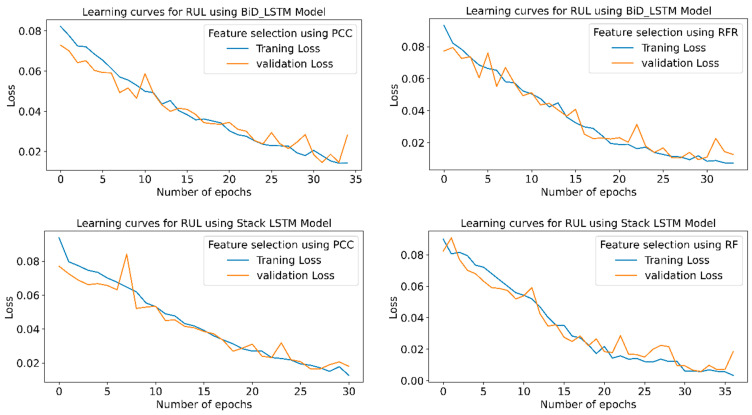
RUL prediction learning curves using CWT-based feature extraction for different LSTM variants.

Figure 21 shows the learning curves for all six conditions of the model.From the learning curves, it is clear that the model which shows maximum accuracy provides the minimum training and testing losses.

**Figure 22:**
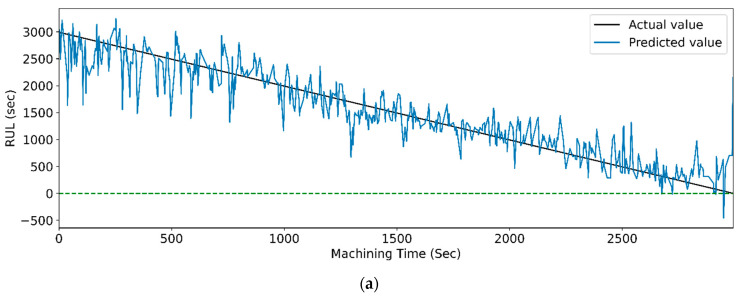

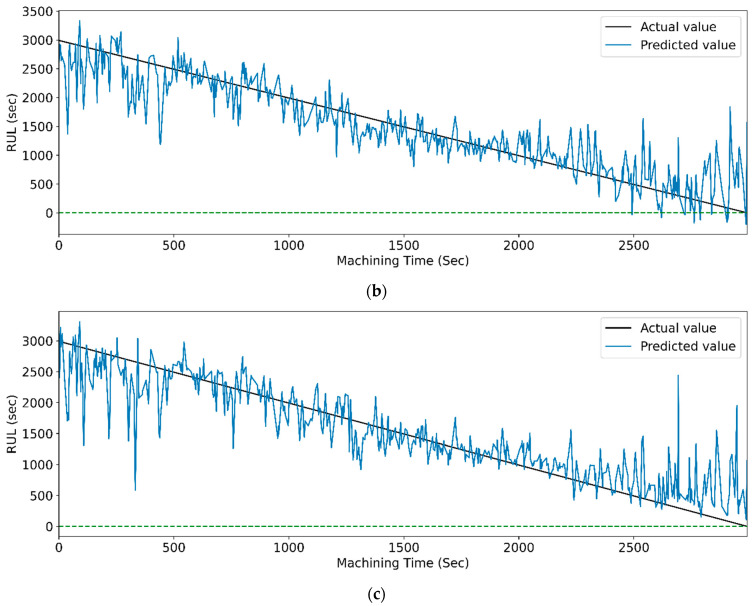
The actual and predicted values of RUL versus machining time for CWT and PCC-based feature selection using different LSTM variants. (**a**) Vanilla, (**b**) Bi-directional, and (**c**) Stack.

**Figure 23:**
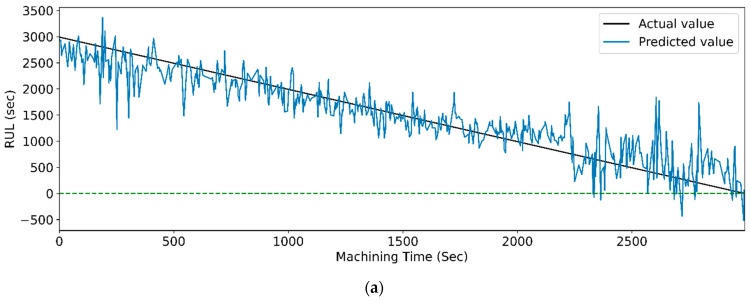

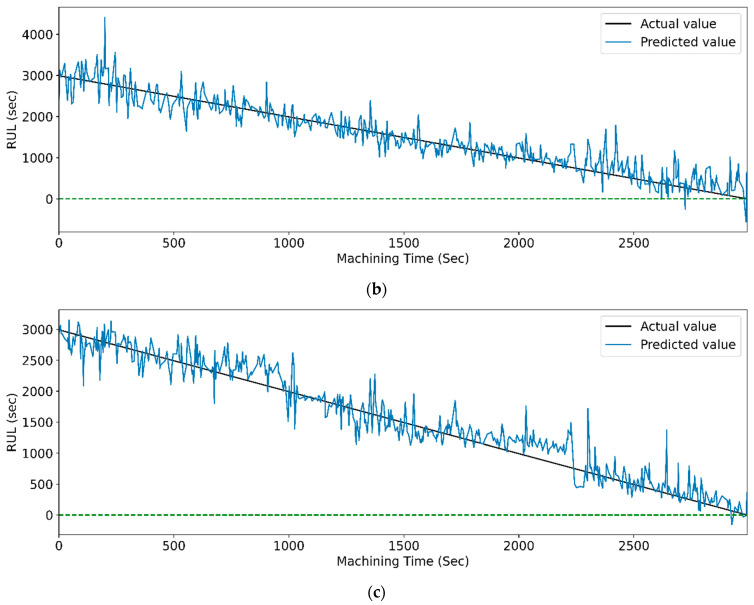
The actual and predicted value of RUL versus machining time for CWT and RFR-based feature selection using different LSTM variants. (**a**) Vanilla, (**b**) Bi-directional, and (**c**) Stack.

Figure 22 and Figure 23 show the graphical representation that indicates the actual and predicted RUL of different LSTM models with respect to machining time. Models with the highest accuracies for both feature selection methods demonstrate the least deviation between the real and anticipated values of RUL concerning machining time.

Similarly, Table 18 shows the performance evaluation parameters of the CNN and CNN-LSTM variants models using CWT time–frequency-based feature extraction techniques for RUL prediction. For the PCC-based feature selection technique, the CNN-Bidirectional-LSTM shows the maximum testing accuracy of 0.960, with 0.051 and 3.576% as RMSE and MAPE values, respectively. In RFR feature selection, CNN-bidirectional LSTM provides a maximum R^2^ score of 0.971 as testing accuracy with 0.048 RMSE value and 3.428% MAPE value.

**Figure 24:**
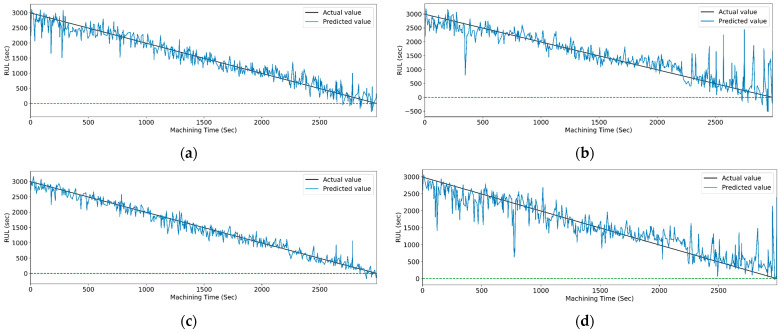
The actual and predicted value of RUL versus machining time for CWT and PCC-based feature selection using different models. (**a**) CCN, (**b**) CNN-LSTM, (**c**) CNN-bidirectional LSTM, and (**d**) CNN-Stack LSTM.

**Figure 25:**
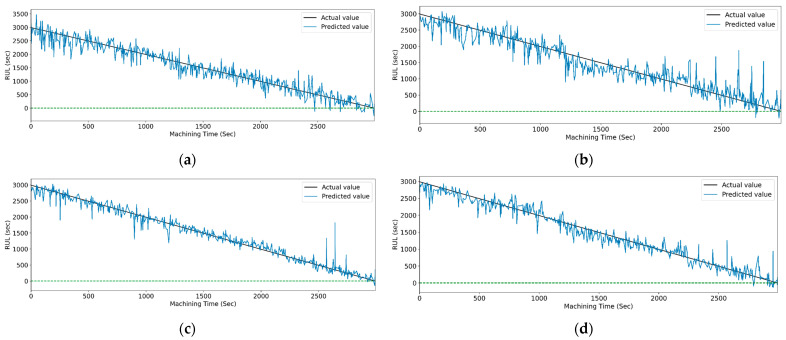
The actual and predicted value of RUL versus machining time for CWT and RFR-based feature selection using different models. (**a**) CCN, (**b**) CNN-LSTM, (**c**) CNN-bidirectional LSTM, and (**d**) CNN-Stack LSTM.

Figure 24 and Figure 25 show the actual and predicted values of the RUL for the PCC-based and RF-based feature selection techniques, respectively, for extracted features using the CWT method. In PCC-based feature selection, Figure 24c shows the minimum deviation between the actual and predicted RUL values, as the CNN-bidirectional-LSTM shows the maximum accuracy. Similarly, in RFR-based feature selection, Figure 25c, the CNN-bidirectional-LSTM shows the minimum deviation in actual and predicted RUL values with a maximum R-squared value of 0.96.

#### 5.3.3. RUL Prediction Using WPT Feature Extraction Technique

The WPT is used to estimate the RUL of the cutting tool. Table 19 shows the performance evaluation parameters of the different LSTM variants model using WPT time–frequency-based feature extraction techniques for RUL prediction. For the PCC-based feature selection technique, the stack LSTM shows the maximum testing accuracy of 0.857, with 0.102 and 7.140% as RMSE and MAPE values, respectively. In RFR feature selection, stack LSTM provides a maximum R^2^ score value of 0.978 as train accuracy and 0.967 as testing accuracy with 0.051 RMSE value and 03.676% MAPE value.

**Figure 26:**
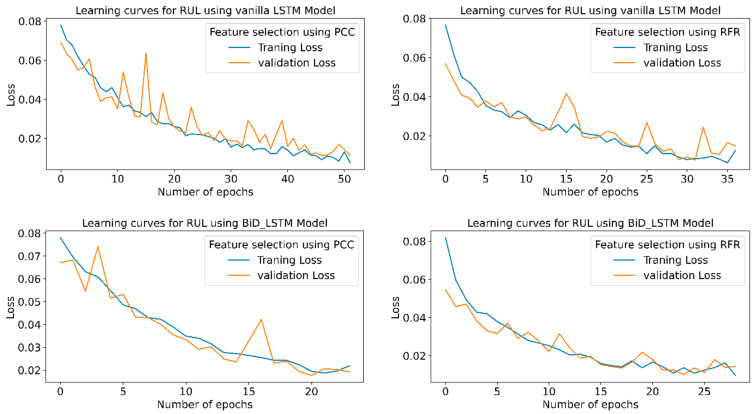

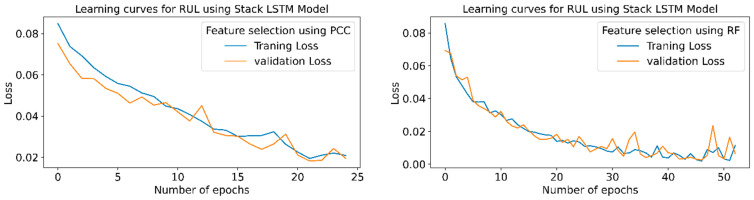
RUL prediction learning curves using WPT-based feature extraction for different LSTM variants.

Figure 26 indicates the training and validation loss learning curves for the prediction of RUL employing various LSTM variants for PCC and RFR-based feature selection techniques. In PCC-based feature selection, vanilla LSTM shows minimum training losses at 51 epochs. The model uses early stopping to avoid overfitting using a three-patient level in the call-back function. At the same time, in RFR based feature selection technique, the Stack LSTM shows minimum training losses at the 52 epochs and indicates the maximum accuracy for the same epochs.

**Figure 27:**
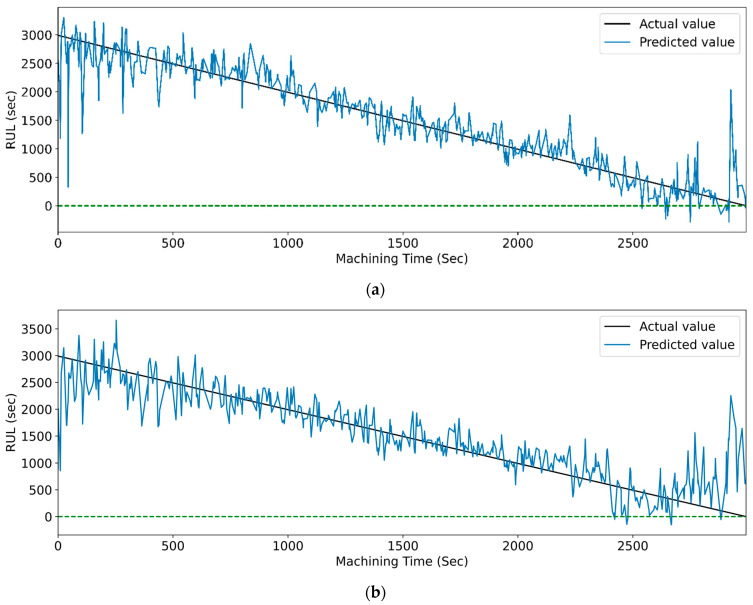

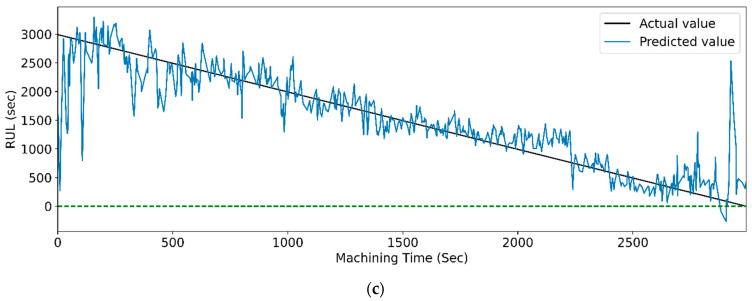
The actual and predicted values of RUL versus machining time for WPT and PCC-based feature selection using different LSTM variants. (**a**) Vanilla, (**b**) Bi-directional, and (**c**) Stack.

**Figure 28:**
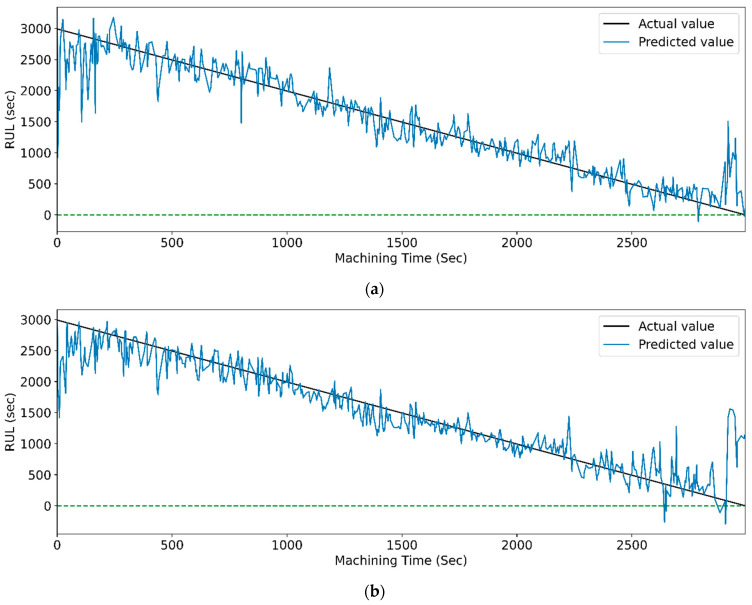

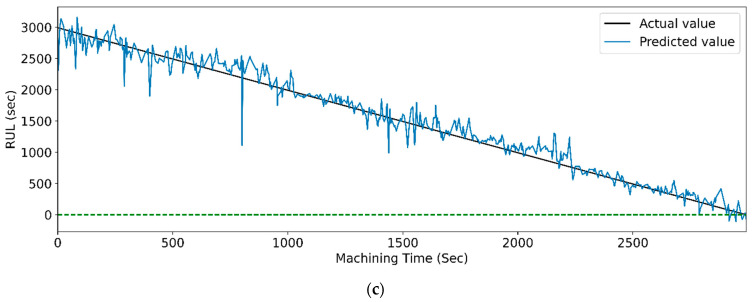
The Actual and predicted values of RUL versus machining time for WPT and RFR-based feature selection using different LSTM variants. (**a**) Vanilla, (**b**) Bi-directional, and (**c**) Stack.

Figure 27 and Figure 28 show the actual and predicted RUL vs. machining time of the cutting tool using the WPT feature extraction technique for PCC and RFR-based feature selection, respectively, for different LSTM variants. From the graphical representation, it is clear that the model with the highest prediction accuracy shows a slight variation between the real and predicted RUL. Figure 14, vanilla LSTM, shows the minimum deviation between the actual and predicted RUL. Whereas, in Figure 15, stack LSTM shows the lowest variation between real and predicted RUL values.

Similarly, Table 20 shows the performance evaluation parameters of the CNN and CNN-LSTM variants models using WPT time–frequency-based feature extraction techniques for RUL prediction. For the PCC-based feature selection technique, the CNN-Bidirectional-LSTM shows the maximum testing accuracy of 0.908, with 0.086 and 5.90% as RMSE and MAPE values, respectively. In RFR feature selection, CNN-bidirectional-LSTM provides a maximum R^2^ score of 0.955 as testing accuracy with 0.056 RMSE value and 03.59% MAPE value.

**Figure 29:**
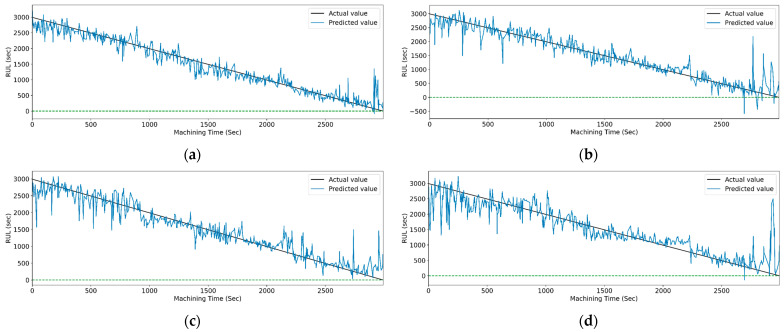
The actual and predicted value of RUL versus machining time for WPT and PCC-based feature selection using different models. (**a**) CCN, (**b**) CNN-LSTM, (**c**) CNN-bidirectional LSTM, and (**d**) CNN-Stack LSTM.

**Figure 30:**
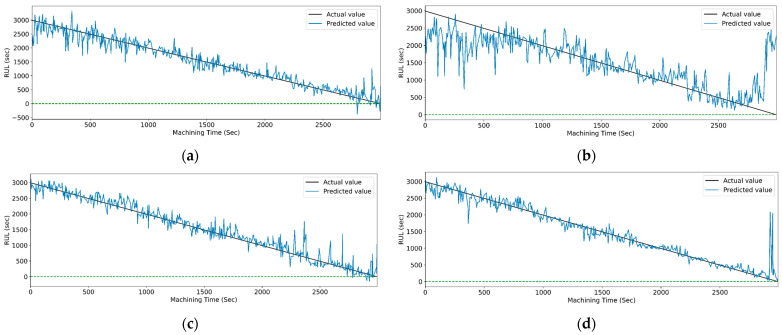
The actual and predicted value of RUL versus machining time for WPT and RFR-based feature selection using different models. (**a**) CCN, (**b**) CNN-LSTM, (**c**) CNN-bidirectional LSTM, and (**d**) CNN-Stack LSTM.

Figure 29 and Figure 30 show the graphical representation of actual and predicted values of the RUL for the PCC-based and RF-based feature selection techniques, respectively, for extracted features using the WPT method. In PCC-based feature selection, Figure 29c shows the minimum deviation between the actual and predicted RUL values, as the CNN-bidirectional-LSTM shows the maximum accuracy. Similarly, as shown in Figure 30d, RFR-based feature selection, the CNN-bidirectional-LSTM, shows the minimum deviation in actual and predicted RUL values.

Table 21 summarizes all the results from the prediction models, including LSTM, LSTM variants, CNN, and CNN with LSTM variants for case W1. In the STFT feature extraction technique, the CNN-stack-LSTM provides the maximum R^2^ value of 0.951 using the RF feature selection technique. In CWT feature extraction, the CNN-bidirectional LSTM provides a maximum R^2^ value of 0.971. In the WPT feature extraction technique, stack-LSTM provides the maximum R^2^ value of 0.967.

Similarly, the model performance is verified on case W2. In the case of W2, 18 runs are required to reach the maximum tool wear value of 0.30 mm. The DL models for RUL predictions provide good results, as summarized in Table 22.

The results show that the RF feature selection technique performs slightly better than the PCC-based feature selection technique. Tool wear, or RUL, is a non-linear and complex phenomenon. The PCC feature selection technique provides better results for linear relationships than non-linear ones. The RF feature selection technique gives better results for non-linear relationships and complex models. In the case of RUL prediction models, ML models show poor prediction performance as the model struggles to capture complex and non-linear relationships in the cutting tool RUL data. In comparison, the DL models show fairly good prediction results in RUL prediction. In this work, based on the results, it is observed that, compared to the normal CCN and LSTM models, LSTM variants and hybrid models (CNN with LSTM variants) provide better results. The LSTM variants and CNN with LSTM variants easily and more accurately understand the temporal or time-related aspects of sequential or time series signals captured for RUL prediction of cutting tool.

## 6. Conclusions

In this work, the IEEE NUAA Ideahouse dataset is used for the cutting tool’s remaining useful life (RUL) prediction. Time–frequency feature extraction techniques such as STFT and WT are used to avoid the limitations of TD and FD feature extraction. The model prediction results are verified using the two cases (W1 and W2) from the dataset. The following conclusions are drawn from the obtained results:

- The RF feature selection technique performs slightly better than the PCC-based feature selection technique. The RF feature selection technique gives better results for non-linear relationships and complex models;
- The DL models such as LSTM, LSTM variants, CNN, and CNN with LSTM variants provide better prediction accuracies than ML models, as these models are effective for the time-series and complex non-linear cutting tool data for RUL estimation;
- In STFT, CWT, and WPT feature extraction techniques, the highest value of R^2^ score is more than 0.95 for LSTM variants and hybrid (CNN with LSTM variants) prediction models;
- The result shows that the TFD feature extraction technique is effective for RUL prediction with deep learning models such as LSTM, LSTM variants, CNN, and hybrid model CNN with LSTM variants.

## Figures and Tables

**Table 1:** Signal acquisition equipment and sampling frequency.

Signal Category	Acquisition Equipment	Sample Frequency (Hz)
Spindle current and power	PLC	300
Vibration	PCB^TM^-W356B11	400
Cutting force	Spike^TM^ sensory tool holder	600

**Table 2:** Details of the orthogonal experiment of IEEE NUAA Ideahouse.

No. of Cases	Feed Per Tooth (mm/r)	Spindle Speed (r/min)	Axial Cutting Depth (mm)	Tool Material	Workpiece Material
W1	0.045	1750	2.5	Solid carbide	TC4
W2	0.045	1800	3		
W3	0.045	1850	3.5		
W4	0.05	1750	3		
W5	0.05	1800	3.5		
W6	0.05	1850	2.5		
W7	0.055	1750	3.5		
W8	0.055	1800	2.5		
W9	0.055	1850	3		

**Table 3:** Tool wear labels for W1 case.

Sr. No.	Flank Wear (mm)				Sr. No	Flank Wear (mm)			
	Flute-1	Flute-2	Flute-3	Flute-4		Flute-1	Flute-2	Flute-3	Flute-4
1	0.05	0.12	0.1	0.05	16	0.17	0.23	0.21	0.14
2	0.1	0.14	0.1	0.05	17	0.18	0.23	0.21	0.14
3	0.12	0.14	0.11	0.09	18	0.18	0.23	0.21	0.15
4	0.12	0.15	0.13	0.1	19	0.18	0.23	0.21	0.15
5	0.13	0.16	0.15	0.1	20	0.19	0.23	0.21	0.15
6	0.13	0.18	0.16	0.1	21	0.19	0.24	0.22	0.15
7	0.14	0.18	0.16	0.1	22	0.19	0.24	0.22	0.15
8	0.15	0.18	0.16	0.12	23	0.19	0.24	0.22	0.15
9	0.16	0.19	0.17	0.12	24	0.19	0.24	0.22	0.15
10	0.16	0.2	0.18	0.12	25	0.19	0.25	0.24	0.15
11	0.16	0.21	0.18	0.12	26	0.19	0.25	0.25	0.15
12	0.17	0.21	0.19	0.13	27	0.2	0.25	0.25	0.15
13	0.17	0.22	0.2	0.13	28	0.2	0.26	0.26	0.15
14	0.17	0.22	0.21	0.13	29	0.2	0.26	0.26	0.15
15	0.17	0.22	0.21	0.14	30	0.2	0.27	0.26	0.15

**Table 4:** The statistical features and their formulae.

Sr. No.	Statistical Features	Formula
1	Mean	$S_{mean=}\frac{1}{N}\sum_{i=1}^{N}s_{i}$
2	Standard deviation	$S_{std}=\sqrt{\sum_{i=1}^{n}{\frac{\left(s_{i}-s_{m}\right)}{\left(n-1\right)}}^{2}}$
3	Variance	$S_{var}=\sum_{i=1}^{n}{\frac{\left(s_{i}-s_{m}\right)}{\left(n-1\right)}}^{2}$
4	Kurtosis	$S_{kur}=\frac{\sum_{i=1}^{N}{\left(s_{i}-s_{m}\right)}^{4}}{(N-1){S_{\sigma }}^{4}}$
5	Skewness	$S_{skew}=\frac{\sum_{i=1}^{n}{\left(s_{i}-s_{m}\right)}^{3}}{(n-1){S_{\sigma }}^{3}}$
6	Root mean square	$S_{rms}=\sqrt{\frac{1}{n}\sum_{i=1}^{n}{s_{i}}^{2}}$
7	Peak to Peak	$S_{peak}=max\left(s_{i}\right)-min\left(s_{i}\right)$
8	Peak amplitude	$S_{CF}=\max\left(s_{i}\right)-(\frac{1}{N}\sum_{i=1}^{N}x_{i})$

**Table 5:** Model performance parameters.

Sr. No.	Performance Parameters	Formula
1	R-squared (R^2^)	$R^{2}=1-\frac{\sum_{i}^{n}{\left({\ddot{a}}_{i}-a_{i}\right)}^{2}}{\sum_{i}^{n}{\left(a_{i}\right)}^{2}}$
2	RMSE	$RMSE=\sqrt{\frac{1}{n}\sum_{i}^{n}{\left({\ddot{a}}_{i}-a_{i}\right)}^{2}}$
3	MAPE	$MAPE=\frac{1}{n}\sum_{i=1}^{n}\left|\frac{\left(a_{i}-{\ddot{a}}_{i}\right)}{a_{i}}\right|$

**Table 6:** Feature extraction and its feature count.

Feature Extraction Method	Feature Count
STFT	64
CWT	64
WPT	128

**Table 7:** Selected features using PCC from STFT feature extraction technique.

Feature Count	Feature	PCC	Feature Count	Feature	PCC
1	stft_rms_Bending_Moment_Y	0.563	12	stft_p2p_vib_x	0.290
2	stft_std_Bending_Moment_Y	0.500	13	stft_peak_amp_vib_x	0.290
3	stft_mean_Bending_Moment_Y	0.499	14	stft_skew_Bending_Moment_Y	0.282
4	stft_var_Bending_Moment_Y	0.498	15	stft_rms_vib_x	0.274
5	stft_p2p_Bending_Moment_Y	0.493	16	stft_kurtosis_Bending_Moment_Y	0.270
6	stft_peak_amp_Bending_Moment_Y	0.493	17	stft_std_Bending_Moment_X	0.256
7	stft_mean_Torsion_Z	0.348	18	stft_var_Bending_Moment_X	0.255
8	stft_skew_Torsion_Z	0.327	19	stft_peak_amp_Bending_Moment_X	0.253
9	stft_kurtosis_Torsion_Z	0.317	20	stft_p2p_Bending_Moment_X	0.253
10	stft_var_vib_x	0.300	21	stft_rms_Bending_Moment_X	0.243
11	stft_std_vib_x	0.297			

**Table 8:** Selected features using RF from STFT feature extraction technique.

Feature Count	Features	Weight	Feature Count	Score	Weight
1	stft_rms_Bending_Moment_Y	32.87	17	stft_peak_amp_Torsion_Z	1.43
2	stft_mean_Torsion_Z	7.13	18	stft_var_vib_x	1.39
3	stft_skew_Torsion_Z	4.37	19	stft_var_vib_y	1.25
4	stft_peak_amp_vib_x	4.15	20	stft_mean_Axial_Force	1.15
5	stft_p2p_vib_x	4.01	21	stft_var_Torsion_Z	0.80
6	stft_mean_Bending_Moment_Y	3.83	22	stft_std_Torsion_Z	0.74
7	stft_std_vib_x	3.20	23	stft_var_Bending_Moment_Y	0.66
8	stft_var_Axial_Force	2.78	24	stft_kurtosis_Bending_Moment_Y	0.65
9	stft_rms_Axial_Force	2.76	25	stft_skew_Bending_Moment_Y	0.62
10	stft_std_Axial_Force	2.70	26	stft_kurtosis_Torsion_Z	0.61
11	stft_rms_vib_x	2.51	27	stft_mean_vib_y	0.59
12	stft_peak_amp_Axial_Force	2.39	28	stft_rms_vib_y	0.58
13	stft_p2p_vib_y	2.00	29	stft_std_Bending_Moment_Y	0.57
14	stft_p2p_Torsion_Z	1.75	30	stft_std_vib_y	0.55
15	stft_p2p_Axial_Force	1.72	31	stft_rms_Torsion_Z	0.54
16	stft_peak_amp_vib_y	1.59			

**Table 9:** Selected features using PCC from CWT feature extraction technique.

Feature Count	Features	PCC	Feature Count	Features	PCC
1	cwt_rms_Bending_Moment_Y	0.372	7	cwt_p2p_Torsion_Z	0.326
2	cwt_std_Bending_Moment_Y	0.372	8	cwt_var_Torsion_Z	0.322
3	cwt_var_Bending_Moment_Y	0.370	9	cwt_peak_amp_Bending_Moment_Y	0.306
4	cwt_std_Torsion_Z	0.333	10	cwt_p2p_Bending_Moment_Y	0.306
5	cwt_rms_Torsion_Z	0.333	11	cwt_skew_Torsion_Z	0.201
6	cwt_peak_amp_Torsion_Z	0.326			

**Table 10:** Selected features using RF from CWT feature extraction technique.

Feature Count	Features	Weight	Feature Count	Features	Weight
1	cwt_mean_Bending_Moment_Y	23.21	23	cwt_kurtosis_Spindle_current	0.93
2	cwt_mean_Torsion_Z	12.49	24	cwt_skew_Axial_Force	0.92
3	cwt_mean_Bending_Moment_X	7.28	25	cwt_kurtosis_Spindle_power	0.89
4	cwt_rms_Torsion_Z	5.06	26	cwt_peak_amp_Bending_Moment_X	0.89
5	cwt_rms_Bending_Moment_Y	3.40	27	cwt_std_Bending_Moment_X	0.87
6	cwt_skew_Bending_Moment_Y	2.64	28	cwt_kurtosis_Axial_Force	0.82
7	cwt_rms_Bending_Moment_X	2.16	29	cwt_rms_Axial_Force	0.82
8	cwt_skew_Torsion_Z	1.95	30	cwt_skew_Spindle_power	0.82
9	cwt_kurtosis_Bending_Moment_X	1.76	31	cwt_var_Bending_Moment_X	0.80
10	cwt_var_Bending_Moment_Y	1.62	32	cwt_skew_Spindle_current	0.73
11	cwt_std_Torsion_Z	1.57	33	cwt_p2p_Bending_Moment_X	0.73
12	cwt_std_Bending_Moment_Y	1.53	34	cwt_kurtosis_vib_x	0.71
13	cwt_var_Torsion_Z	1.47	35	cwt_peak_amp_Axial_Force	0.69
14	cwt_mean_Axial_Force	1.37	36	cwt_std_Axial_Force	0.69
15	cwt_kurtosis_Torsion_Z	1.30	37	cwt_var_Axial_Force	0.69
16	cwt_kurtosis_Bending_Moment_Y	1.27	38	cwt_skew_vib_y	0.67
17	cwt_skew_Bending_Moment_X	1.16	39	cwt_mean_Spindle_current	0.67
18	cwt_peak_amp_Bending_Moment_Y	1.05	40	cwt_mean_Spindle_power	0.66
19	cwt_p2p_Torsion_Z	1.04	41	cwt_p2p_Axial_Force	0.66
20	cwt_skew_vib_x	1.02	42	cwt_mean_vib_y	0.59
21	cwt_peak_amp_Torsion_Z	0.94	43	cwt_p2p_Spindle_current	0.51
22	cwt_p2p_Bending_Moment_Y	0.93			

**Table 11:** Selected features using PCC from WPT feature extraction technique.

Feature Count	Features	PCC	Feature Count	Features	PCC
1	a_rms_Bending_Moment_Y	0.66	14	a_peak_amp_Bending_Moment_Y	0.36
2	a_skew_Torsion_Z	0.60	15	a_p2p_Bending_Moment_Y	0.36
3	a_mean_Bending_Moment_Y	0.57	16	d_p2p_Torsion_Z	0.34
4	a_std_Bending_Moment_Y	0.50	17	d_peak_amp_Torsion_Z	0.34
5	a_var_Bending_Moment_Y	0.50	18	d_rms_Torsion_Z	0.34
6	d_rms_Bending_Moment_Y	0.47	19	d_std_Torsion_Z	0.34
7	d_std_Bending_Moment_Y	0.47	20	d_var_Torsion_Z	0.34
8	d_var_Bending_Moment_Y	0.47	21	a_kurtosis_vib_x	0.31
9	d_peak_amp_Bending_Moment_Y	0.40	22	a_kurtosis_vib_y	0.29
10	d_p2p_Bending_Moment_Y	0.40	23	a_peak_amp_Torsion_Z	0.27
11	a_std_Torsion_Z	0.37	24	a_p2p_Torsion_Z	0.27
12	a_var_Torsion_Z	0.37	25	a_mean_Bending_Moment_X	0.25
13	a_skew_Bending_Moment_Y	0.36	26	a_rms_Bending_Moment_X	0.25

**Table 12:** Selected features using RF from WPT feature extraction technique.

Feature Count	Feature	Weight	Feature Count	Feature	Weight
1	a_rms_Bending_Moment_Y	32.88	11	a_mean_vib_y	1.35
2	a_skew_Bending_Moment_Y	23.38	12	a_p2p_Torsion_Z	1.30
3	a_skew_Torsion_Z	4.60	13	a_peak_amp_Torsion_Z	1.26
4	a_rms_vib_x	4.58	14	a_p2p_Bending_Moment_Y	0.74
5	a_mean_vib_x	3.36	15	a_mean_Torsion_Z	0.72
6	a_rms_Axial_Force	3.22	16	a_var_Bending_Moment_Y	0.67
7	a_kurtosis_Torsion_Z	3.18	17	a_peak_amp_Bending_Moment_Y	0.65
8	a_mean_Axial_Force	2.48	18	a_mean_Bending_Moment_Y	0.61
9	a_kurtosis_Bending_Moment_Y	2.18	19	a_std_Bending_Moment_Y	0.59
10	a_rms_vib_y	1.36			

**Table 13:** RUL prediction for PCC-based feature selection techniques using different ML models.

Feature Extraction Techniques	Prediction Models	RUL Prediction		
		Performance Evaluation on Testing Data		
		R^2^	RMSE	MAPE
STFT	SVR	0.235	0.249	17.983
	RFR	0.363	0.227	16.001
	GBR	0.316	0.235	17.871
CWT	SVR	0.062	0.288	22.631
	RFR	0.100	0.270	20.900
	GBR	0.084	0.273	21.922
WPT	SVR	0.216	0.252	18.344
	RFR	0.366	0.227	15.933
	GBR	0.320	0.234	17.510

**Table 14:** RUL prediction for RFR-based feature selection techniques using different ML models.

Feature Extraction Techniques	Prediction Models	RUL Prediction		
		Performance Evaluation on Testing Data		
		R^2^	RMSE	MAPE
STFT	SVR	0.241	0.248	18.447
	RFR	0.383	0.224	16.001
	GBR	0.346	0.230	17.406
CWT	SVR	0.081	0.289	22.820
	RFR	0.102	0.270	21.111
	GBR	0.111	0.269	21.856
WPT	SVR	0.347	0.230	16.045
	RFR	0.496	0.2026	13.495
	GBR	0.452	0.211	15.194

**Table 15:** STFT feature extraction-based RUL prediction for PCC and RFR feature selection techniques using different LSTM variants.

Feature Selection Techniques	Prediction Models	RUL Prediction					
		Performance Evaluation on Training Data			Performance Evaluation on Testing Data		
		R^2^	RMSE	MAPE	R^2^	RMSE	MAPE
Pearson’s Correlation Coefficient (PCC)	Vanilla LSTM	0.741	0.147	10.163	0.706	0.152	10.523
	Bi-direction LSTM	0.800	0.129	08.807	0.755	0.139	09.175
	Stack LSTM	0.865	0.106	06.743	0.802	0.125	07.372
Random Forest Regressor (RFR)	Vanilla LSTM	0.780	0.135	08.998	0.737	0.144	09.510
	Bi-direction LSTM	0.704	0.157	10.715	0.654	0.165	11.461
	Stack LSTM	0.809	0.126	08.138	0.782	0.131	08.520

**Table 16:** STFT feature extraction-based RUL prediction for PCC and RFR feature selection techniques using CNN and CNN-LSTM variants.

Feature Selection Techniques	Prediction Models	RUL Prediction					
		Performance Evaluation on Training Data			Performance Evaluation on Testing Data		
		R^2^	RMSE	MAPE	R^2^	RMSE	MAPE
Pearson’s Correlation Coefficient (PCC)	CNN	0.878	0.101	07.057	0.775	0.133	08.946
	CNN-LSTM	0.934	0.074	05.426	0.881	0.097	06.877
	CNN-Bi-LSTM	0.788	0.133	08.605	0.753	0.140	09.090
	CNN-Stack-STM	0.870	0.104	06.842	0.833	0.115	07.421
Random Forest Regressor (RFR)	CNN	0.972	0.048	03.570	0.930	0.074	05.499
	CNN-LSTM	0.829	0.119	08.067	0.774	0.134	08.728
	CNN-Bi-LSTM	0.906	0.088	05.891	0.838	0.113	07.090
	CNN-Stack-LSTM	0.964	0.054	03.681	0.951	0.062	04.161

**Table 17:** CWT feature extraction-based RUL prediction for PCC and RFR feature selection techniques using different LSTM variants.

Feature Selection Techniques	Prediction Models	RUL Prediction					
		Performance Evaluation on Training Data			Performance Evaluation on Testing Data		
		R^2^	RMSE	MAPE	R^2^	RMSE	MAPE
Pearson’s Correlation Coefficient (PCC)	Vanilla LSTM	0.907	0.087	06.503	0.851	0.104	07.359
	Bi-direction LSTM	0.864	0.106	07.505	0.818	0.120	08.446
	Stack LSTM	0.861	0.107	07.506	0.793	0.128	08.577
Random Forest Regressor (RFR)	Vanilla LSTM	0.882	0.099	07.176	0.838	0.113	08.369
	Bi-direction LSTM	0.909	0.087	06.582	0.881	0.097	07.274
	Stack LSTM	0.953	0.062	04.789	0.927	0.075	05.781

**Table 18:** CWT feature extraction-based RUL prediction for PCC and RFR feature selection techniques using CNN and CNN-LSTM variants.

Feature Selection Techniques	Prediction Models	RUL Prediction					
		Performance Evaluation On Training Data			Performance Evaluation on Testing Data		
		R^2^	RMSE	MAPE	R^2^	RMSE	MAPE
Pearson’s Correlation Coefficient (PCC)	CNN	0.981	0.039	03.157	0.934	0.072	05.301
	CNN-LSTM	0.937	0.072	05.236	0.858	0.106	06.922
	CNN-Bi-LSTM	0.987	0.0317	02.447	0.960	0.051	03.576
	CNN Stack LSTM	0.900	0.091	06.516	0.828	0.117	07.814
Random Forest Regressor (RFR)	CNN	0.962	0.055	04.415	0.919	0.080	06.177
	CNN-LSTM	0.935	0.075	05.729	0.890	0.093	06.880
	CNN-Bi-LSTM	0.992	0.025	01.95	0.971	0.048	03.428
	CNN-Stack-LSTM	0.976	0.044	03.418	0.953	0.059	04.354

**Table 19:** WPT feature extraction-based RUL prediction for PCC and RFR feature selection techniques using different LSTM variants.

Feature Selection Techniques	Prediction Models	RUL Prediction					
		Performance Evaluation on Training Data			Performance Evaluation on Testing Data		
		R^2^	RMSE	MAPE	R^2^	RMSE	MAPE
Pearson’s Correlation Coefficient (PCC)	Vanilla LSTM	0.922	0.080	06.021	0.857	0.102	07.140
	Bi-direction LSTM	0.814	0.124	08.576	0.778	0.132	09.087
	Stack LSTM	0.828	0.119	07.698	0.771	0.134	08.134
Random Forest Regressor (RFR)	Vanilla LSTM	0.937	0.072	04.914	0.901	0.088	05.721
	Bi-direction LSTM	0.897	0.092	05.966	0.873	0.100	06.533
	Stack LSTM	0.978	0.042	03.043	0.964	0.051	03.676

**Table 20:** WPT feature extraction-based RUL prediction for PCC and RFR feature selection techniques using CNN and CNN-LSTM variants.

Feature Selection Techniques	Prediction Models	RUL Prediction					
		Performance Evaluation on Training Data			Performance Evaluation on Testing Data		
		R^2^	RMSE	MAPE	R^2^	RMSE	MAPE
Pearson’s Correlation Coefficient (PCC)	CNN	0.979	0.041	03.192	0.941	0.068	04.943
	CNN-LSTM	0.946	0.066	05.023	0.903	0.087	05.925
	CNN -Bi-LSTM	0.950	0.064	04.700	0.908	0.086	05.905
	CNN-Stack-LSTM	0.878	0.100	06.915	0.827	0.117	07.630
Random Forest Regressor (RFR)	CNN	0.966	0.052	03.869	0.926	0.076	05.522
	CNN-LSTM	0.630	0.175	11.452	0.946	0.051	05.522
	CNN-Bi-LSTM	0.979	0.041	03.211	0.948	0.064	04.647
	CNN-Stack-LSTM	0.977	0.043	0.030	0.955	0.058	03.590

**Table 21:** Summarized performance evaluation for different feature extraction techniques and DL models for RUL prediction using PCC and RFR-based feature selection techniques (Case-W1).

Feature Extraction Techniques	Prediction Models	RUL Prediction					
		Feature Selection Using Pearson’s Correlation Coefficient (PCC)			Feature Selection Using Random Forest (RF)		
		R^2^	RMSE	MAPE	R^2^	RMSE	MAPE
STFT	Vanilla LSTM	0.706	0.152	10.523	0.737	0.144	09.510
	Bi-direction LSTM	0.755	0.139	09.175	0.654	0.165	11.461
	Stack LSTM	0.802	0.125	07.372	0.782	0.131	08.520
	CNN	0.775	0.133	08.946	0.930	0.074	05.499
	CNN-LSTM	0.881	0.097	06.877	0.774	0.134	08.728
	CNN-Bi-LSTM	0.753	0.140	09.090	0.838	0.113	07.090
	CNN-Stack-LSTM	0.833	0.115	07.421	0.951	0.062	04.161
CWT	Vanilla LSTM	0.851	0.104	07.359	0.838	0.113	08.369
	Bi-direction LSTM	0.818	0.120	08.446	0.881	0.097	07.274
	Stack LSTM	0.793	0.128	08.577	0.927	0.075	05.781
	CNN	0.934	0.072	05.301	0.919	0.080	06.177
	CNN-LSTM	0.858	0.106	06.922	0.890	0.093	06.880
	CNN-Bi-LSTM	0.960	0.051	03.576	0.971	0.048	03.428
	CNN-Stack-LSTM	0.828	0.117	07.814	0.953	0.059	04.354
WPT	Vanilla LSTM	0.857	0.102	07.140	0.901	0.088	05.721
	Bi-direction LSTM	0.778	0.132	09.087	0.873	0.100	06.533
	Stack LSTM	0.771	0.134	08.134	0.964	0.051	03.676
	CNN	0.941	0.068	04.943	0.926	0.076	05.522
	CNN-LSTM	0.903	0.087	05.925	0.946	0.065	04.448
	CNN-Bi-LSTM	0.908	0.086	05.905	0.948	0.064	04.647
	CNN-Stack-LSTM	0.827	0.117	07.630	0.955	0.058	03.590

**Table 22:** Summarized performance evaluation for different feature extraction techniques and LSTM variants for RUL prediction using PCC and RFR-based feature selection techniques (Case-W2).

Feature Extraction Techniques	Prediction Models	RUL Prediction					
		Feature Selection Using Pearson’s Correlation Coefficient (PCC)			Feature Selection Using Random Forest Regressor (RFR)		
		R^2^	RMSE	MAPE	R^2^	RMSE	MAPE
STFT	Vanilla LSTM	0.949	0.064	04.647	0.946	0.066	04.780
	Bi-direction LSTM	0.954	0.062	04.652	0.926	0.079	05.767
	Stack LSTM	0.832	0.120	07.369	0.940	0.071	04.866
	CNN	0.962	0.057	03.972	0.956	0.061	04.367
	CNN-LSTM	0.898	0.094	05.803	0.891	0.096	06.967
	CNN-Bi-LSTM	0.904	0.087	05.975	0.967	0.044	03.256
	CNN-Stack-LSTM	0.812	0.127	08.412	0.886	0.099	06.263
CWT	Vanilla LSTM	0.883	0.100	06.506	0.965	0.054	03.867
	Bi-direction LSTM	0.788	0.135	09.306	0.900	0.926	05.950
	Stack LSTM	0.793	0.128	0.085	0.949	0.066	04.197
	CNN	0.927	0.079	05.264	0.929	0.078	05.103
	CNN-LSTM	0.928	0.078	05.684	0.810	0.128	08.679
	CNN-Bi-LSTM	0.972	0.048	03.123	0.979	0.042	02.965
	CNN-Stack-LSTM	0.963	0.056	03.984	0.910	0.087	04.969
WPT	Vanilla LSTM	0.979	0.0421	03.195	0.977	0.044	03.243
	Bi-direction LSTM	0.971	0.048	03.649	0.824	0.118	08.063
	Stack LSTM	0.965	0.054	04.043	0.985	0.034	02.728
	CNN	0.971	0.049	03.672	0.950	0.064	04.700
	CNN-LSTM	0.975	0.045	03.478	0.977	0.043	03.012
	CNN-Bi-LSTM	0.878	0.100	06.915	0.970	0.044	03.032
	CNN-Stack-LSTM	0.827	0.117	07.630	0.955	0.059	03.590

## Data Availability

Not applicable.
